# Computer extracted gland features from H*&*E predicts prostate cancer recurrence comparably to a genomic companion diagnostic test: a large multi-site study

**DOI:** 10.1038/s41698-021-00174-3

**Published:** 2021-05-03

**Authors:** Patrick Leo, Andrew Janowczyk, Robin Elliott, Nafiseh Janaki, Kaustav Bera, Rakesh Shiradkar, Xavier Farré, Pingfu Fu, Ayah El-Fahmawi, Mohammed Shahait, Jessica Kim, David Lee, Kosj Yamoah, Timothy R. Rebbeck, Francesca Khani, Brian D. Robinson, Lauri Eklund, Ivan Jambor, Harri Merisaari, Otto Ettala, Pekka Taimen, Hannu J. Aronen, Peter J. Boström, Ashutosh Tewari, Cristina Magi-Galluzzi, Eric Klein, Andrei Purysko, Natalie NC Shih, Michael Feldman, Sanjay Gupta, Priti Lal, Anant Madabhushi

**Affiliations:** 1 Department of Biomedical Engineering, Case Western Reserve University, Cleveland, OH USA; 2 Department of Oncology, Lausanne University Hospital and Lausanne University, Lausanne, Switzerland; 3 Department of Pathology, University Hospitals Cleveland Medical Center, Cleveland, OH USA; 4 Department of Pathology, Harvard Medical School, Brigham and Women’s Hospital, Boston, MA USA; 5 Public Health Agency of Catalonia, Lleida, Catalonia Spain; 6 Department of Population and Quantitative Health Sciences, Case Western Reserve University, Cleveland, OH USA; 7 Department of Urology, Penn Presbyterian Medical Center, Philadelphia, PA USA; 8 Moffitt Cancer Center, Department of Radiation Oncology, University of South Florida, Tampa, FL USA; 9 T.H. Chan School of Public Health and Dana Farber Cancer Institute, Harvard University, Boston, MA USA; 10 Departments of Pathology and Laboratory Medicine and Urology, Weill Cornell Medicine, New York, NY USA; 11 Department of Pathology, University of Turku, Institute of Biomedicine and Turku University Hospital, Turku, Finland; 12 Department of Diagnostic Radiology, University of Turku, Turku, Finland; 13 Department of Urology, University of Turku, Institute of Biomedicine and Turku University Hospital, Turku, Finland; 14 Turku University Hospital, Medical Imaging Centre of Southwest Finland, Turku, Finland; 15 Department of Urology, University of Turku and Turku University Hospital, Turku, Finland; 16 Department of Urology, Icahn School of Medicine at Mount Sinai, New York, NY USA; 17 Department of Pathology, University of Alabama at Birmingham, Birmingham, AL USA; 18 Cleveland Clinic, Glickman Urological and Kidney Institute, Cleveland, OH USA; 19 Cleveland Clinic, Imaging Institute, Section of Abdominal Imaging, Cleveland, OH USA; 20 Department of Pathology, University of Pennsylvania, Philadelphia, PA USA; 21 Department of Urology, Case Western Reserve University, Cleveland, OH USA; 22 Louis Stokes Cleveland Veterans Administration Medical Center, Cleveland, OH USA

**Keywords:** Prognostic markers, Prostate cancer

## Abstract

Existing tools for post-radical prostatectomy (RP) prostate cancer biochemical recurrence (BCR) prognosis rely on human pathologist-derived parameters such as tumor grade, with the resulting inter-reviewer variability. Genomic companion diagnostic tests such as Decipher tend to be tissue destructive, expensive, and not routinely available in most centers. We present a tissue non-destructive method for automated BCR prognosis, termed "Histotyping", that employs computational image analysis of morphologic patterns of prostate tissue from a single, routinely acquired hematoxylin and eosin slide. Patients from two institutions (*n* = 214) were used to train Histotyping for identifying high-risk patients based on six features of glandular morphology extracted from RP specimens. Histotyping was validated for post-RP BCR prognosis on a separate set of *n* = 675 patients from five institutions and compared against Decipher on *n* = 167 patients. Histotyping was prognostic of BCR in the validation set (*p* < 0.001, univariable hazard ratio [HR] = 2.83, 95% confidence interval [CI]: 2.03–3.93, concordance index [c-index] = 0.68, median years-to-BCR: 1.7). Histotyping was also prognostic in clinically stratified subsets, such as patients with Gleason grade group 3 (HR = 4.09) and negative surgical margins (HR = 3.26). Histotyping was prognostic independent of grade group, margin status, pathological stage, and preoperative prostate-specific antigen (PSA) (multivariable *p* < 0.001, HR = 2.09, 95% CI: 1.40–3.10, *n* = 648). The combination of Histotyping, grade group, and preoperative PSA outperformed Decipher (c-index = 0.75 vs. 0.70, *n* = 167). These results suggest that a prognostic classifier for prostate cancer based on digital images could serve as an alternative or complement to molecular-based companion diagnostic tests.

## Introduction

Tumor morphology is associated with cancer aggressiveness in prostate cancer (PCa). Gleason grading, used by pathologists to score the loss of glandular structure and organization in tissue^1^, is strongly correlated with patient outcome^2^. While Gleason grading is done by pathologists and is therefore subjective^3^, computerized image analysis of tissue can quantitatively define tumor morphology. Quantitative histomorphometric (QH) approaches implicitly capture attributes of tumor grade through features of glandular and nuclear shape^4^, arrangement^5^, or disorder^6^, as well as tissue texture^7^. Characteristics of aggressive PCa, such as poorly formed lumens, can be captured by combinations of these features. Studies have shown an association between QH features and patient outcome^6–10^. However multi-site evaluation has been a challenge for QH approaches, in part due to pre-analytic variation between sites in specimen preparation, staining, and scanning.

Radical prostatectomy (RP), the surgical removal of the prostate, remains the most common curative therapy for PCa^11^. Following RP, some patients will experience biochemical recurrence (BCR), defined by consecutive serum prostate-specific antigen (PSA) test results >0.2 ng/mL. BCR is a surrogate endpoint for prostate cancer and is associated with a hazard ratio (HR) of 4.32^12^ for disease-specific death. In the STAMPEDE trial, adjuvant therapy reduced metastasis and disease-specific death^13^, though adjuvant therapy is not appropriate for all patients due to the low overall mortality rate of PCa^14^. Estimates of a patient’s risk of BCR post-surgery could help identify those patients who might benefit from adjuvant therapy while avoiding unnecessary treatment of low-risk patients. Nomograms, the current gold standard for BCR prognosis, produce a probability of BCR based on clinical variables^15^ but do not provide perfect risk stratification^16^, motivating the development of new assays. In addition to nomograms, prognostic molecular companion diagnostics exist^17–19^, but these are tissue destructive, preventing analysis of the entire tumor or retesting of the same sample, and expensive.

In this study, we present a QH method for BCR prognosis using automated analysis of an H*&*E slide from the dominant tumor nodule. A total of 242 features were extracted from slides of *n* = 889 patients. From the *n* = 214 of these patients used for training, 51 features that were stable across staining and scanner variation were used to construct an elastic-net penalized Cox regression model. The Cox model selected six features associated with high-risk disease and used the weighted sum of these features to estimate the BCR risk for each patient. This model, termed Histotyping, was then validated on *n* = 675 patients. Histotyping was compared to the Decipher genomic classifier in *n* = 167 patient subset. Decipher consists of 22 RNA-expression-based genomic markers that are involved in prostate cancer pathogenesis and have been validated for prognosis of metastasis^18^ and BCR^20,21^.

## Results

### Robustness of Histotyping to site-specific effects

**Fig. 1:**
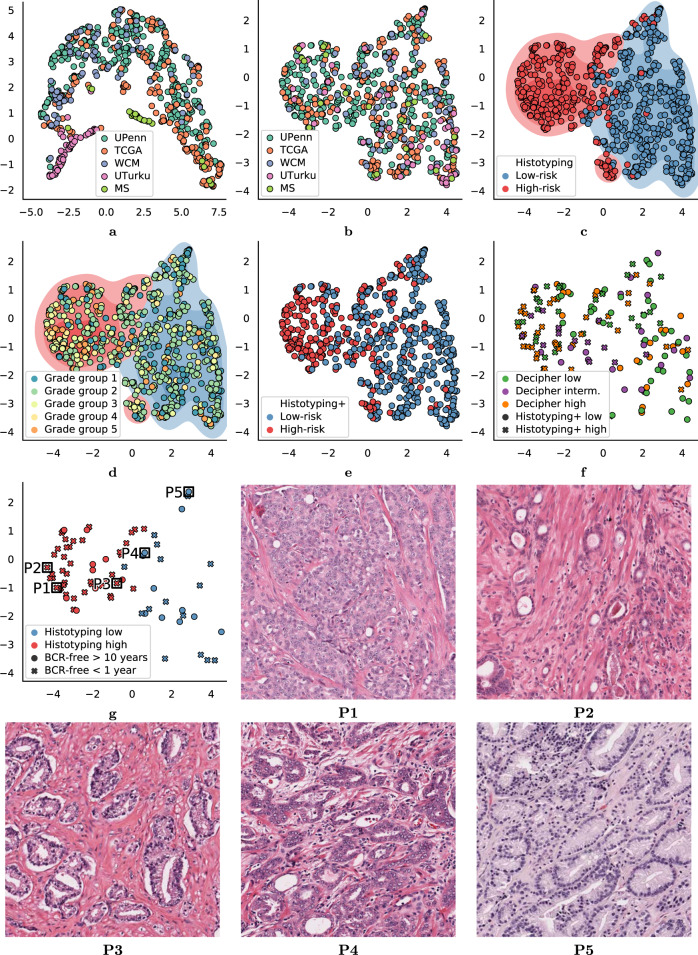
UMAP embedding of image properties and Histotyping features. Shown are UMAP projections based on (**a**) image properties and (**b**–**g**) the six features used in Histotyping on the entire validation set. Each dot represents a patient, and dots are colored according to: **a**, **b** the site the patient originated from, **c** Histotyping risk group, with kernel density, estimates shown for the Histotyping risk groups, **d** Gleason grade group, including kernel density estimates from (**c**), (**e**) Histotyping+ risk group, and (**f**) Decipher score, with markers indicating Histotyping+ risk group. **g** shows the patients who had BCR in less than one year or were BCR-free for more than 10 years, with outcomes indicated by marker shape. Regions of interest from select patients are shown from various locations in (**g**). Patients P1, P2, and P3 were Histotyping high-risk patients who had early BCR, while P4 and P5 were low-risk patients who were BCR free for more than 10 years.

The results of UMAP embedding of the validation set are shown in Fig. 1. While the images from each site tended to segregate in the image-metric embedding, no site formed a distinct cluster in the Histotyping feature space. This suggests that the pathology images varied considerably in brightness, staining, and contrast across the sites, however, the Histotyping features were not adversely affected by variations in staining or scanning across the different laboratories.

### Robustness of Histotyping to annotation perturbations

**Fig. 2:**
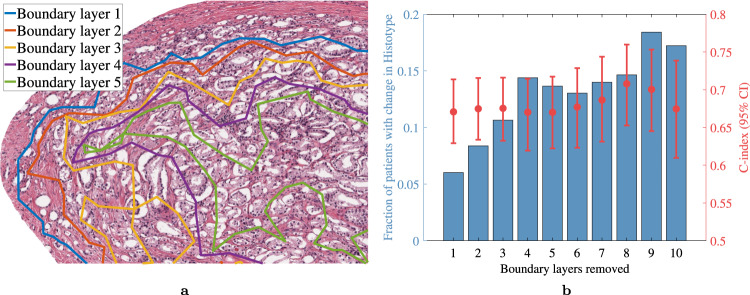
Visualization of the sensitivity analysis experiment. **a** The first five boundary layers of a sample region of interest. In this panel, tissue outside of the original annotation was replaced with white background for clarity. **b** Results of removing an increasing number of boundary layers from the annotations of the validation set patients. The bars depict the fraction of patients who were originally Histotyping low/high-risk who changed to high-/low-risk after removing a given number of boundary layers. The dots show the c-index and 95% CI of Histotyping based on scores calculated after removing boundary layers. These results suggest that Histotyping is robust to variation in pathologist judgment on tumor boundary.

The concordance-index (c-index) of Histotyping remained nearly constant as boundary layers were removed from the annotations (Fig. 2), though 6% of patients had a different risk category when removing a single boundary layer, rising to 11% at three boundary layers. This suggests that Histotyping’s overall performance was relatively consistent across changes in the manually selected region of interest. However, additional study is needed on automated slide and tumor region selection, as well as approaches for aggregating Histotyping feature measurements across multiple cancer foci.

### The prognostic power of histotyping

**Fig. 3:**
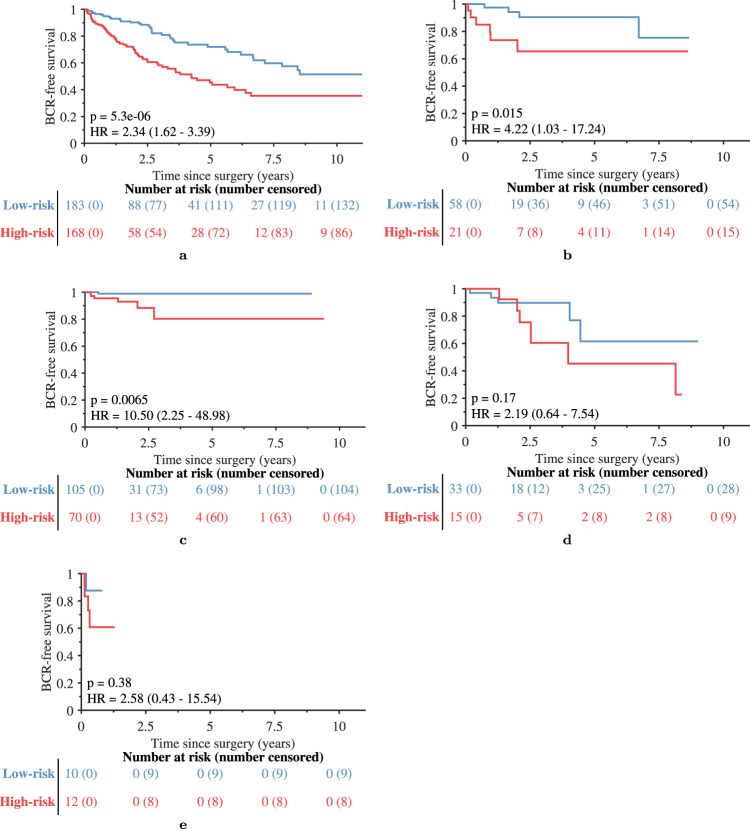
Kaplan-Meier BCR-free survival plots of patients from each site. Patients from **a** UPenn, **b** WCM, **c** TCGA, **d** UTurku, and (**e**) MS are categorized as (blue) low-risk and (red) high-risk.

Histotyping was significantly prognostic of BCR in the training (*p* < 0.001, HR = 2.64, 95% confidence interval [CI]: 1.56–4.44, c-index = 0.63) and validation (*p* < 0.001, HR = 2.83, 95% CI: 2.03–3.93, c-index = 0.68) sets. The features selected by the Cox regression model on the training set are shown in Table 1 and consist of five measures of lumen shape and one feature of lumen arrangement. Histotyping results in each site of the validation cohort are shown in Fig. 3. While there was a separation between Histotyping low-risk and high-risk patients in all five sites, this separation was not significant in the UTurku and MS cohorts, a result potentially influenced by the small number of patients in these sets, with just 48 and 22 patients, respectively.

**Table 1:** The set of 6 gland lumen features selected by the elastic-net Cox regression model as being most prognostic of BCR on the *n* = 214 patient training set.

Feature name	Hazard ratio
Shape: Mean Fourier descriptor 3	1.002
Shape: Standard deviation of the first invariant moment	0.932
Shape: Median mean/max imum ratio of radius	1.100
Shape: 5%/95% Fourier descriptor 6	0.968
Shape: 5%/95% Fourier descriptor 9	0.929
Sub-Graph: Kurtosis of edge length	0.977

The hazard ratio of feature *x* is ${e}^{{\beta }_{x}}$ where *β* is the vector of weights from the fitted Cox regression model. The hazard ratios are shown here reflect the risk of an increase of one standard deviation in feature value on the training set. A hazard ratio less than 1 implies that an increase in that feature’s value is associated with a reduced risk of BCR, while a hazard ratio greater than 1 implies the opposite

**Fig. 4:**
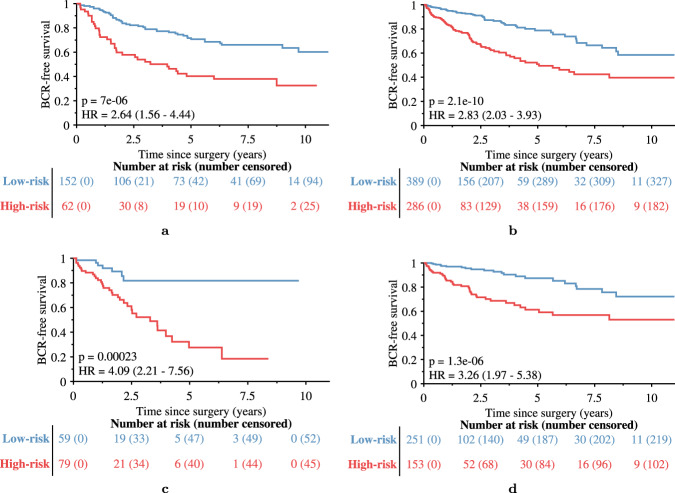
Kaplan-Meier BCR-free survival plots of patients in the training and validation sets. Patients are categorized as (blue) Histotyping low-risk and (red) Histotyping high-risk. Shown are (**a**) the *n* = 214 patient training set, **b** the *n* = 675 patient validation set, (**c**) the *n* = 138 patients of the validation set with Gleason grade group of 3, and (**d**) the *n* = 404 patients of the validation set with negative surgical margins.

As shown in Fig. 4, Histotyping was prognostic in patients with (a) Gleason grade group 3 (HR = 4.09) and (b) negative surgical margins (HR = 3.26). In total, 15 clinically stratified groups were tested (low and high age, preoperative PSA, tumor stage, positive and negative surgical margins, patient Caucasian-American and African-American race, and each Gleason grade group). Bonferroni correction for multiple hypothesis testing yielded a corrected significance threshold of 0.05/15 = 0.0033. The Gleason grade group 3 and negative surgical margin subcohorts had *p*-values below this threshold, as did all other cohorts with the exception of some Gleason grade groups. Results in every subcohort are available in the supplementary information. Histotyping was prognostic independent of common clinical markers both as a continuous score (*p* = 0.002, HR = 1.17, 95% CI: 1.06–1.28) and as a categorical low/high-risk grouping (*p* < 0.001, HR = 2.09, 95% CI: 1.40–3.10), shown in Table 2.

**Table 2:** Cox proportional hazard univariable (UVA) and multivariable (MVA) analysis of BCR including the Histotyping risk score with Gleason grade group, margin status, preoperative PSA, and pathological tumor stage in *n* = 648 patients of the validation set with available clinical information.

	UVA		MVA (Histotyping continuous)		MVA (Histotyping categorical)	
Variable	Hazard ratio (95% CI)	*p*	Hazard ratio (95% CI)	*p*	Hazard ratio (95% CI)	*p*
Histotyping (increase of 0.1)	1.23 (1.13–1.34)	<0.001	1.17 (1.06–1.28)	0.002	–	
Histotyping low-risk	ref		–		ref	
Histotyping high-risk	2.40 (1.64–3.52)	<0.001	–		2.09 (1.40–3.10)	<0.001
Gleason grade group 1	ref		ref		ref	
Gleason grade group 2	1.41 (0.79–2.50)	0.246	0.97 (0.54–1.77)	0.931	0.97 (0.53–1.76)	0.916
Gleason grade group 3	3.07 (1.65–5.71)	<0.001	1.48 (0.76–2.88)	0.254	1.50 (0.77–2.90)	0.234
Gleason grade group 4	5.29 (2.58–10.85)	<0.001	3.33 (1.55–7.14)	0.002	3.44 (1.61–7.34)	0.001
Gleason grade group 5	6.68 (3.27–13.63)	<0.001	2.69 (1.28–5.64)	0.009	2.83 (1.35–5.89)	0.006
Positive surgical margins	2.34 (1.55–3.54)	<0.001	1.29 (0.82–2.02)	0.271	1.26 (0.80–1.99)	0.312
Log2 preoperative PSA, ng/mL	1.77 (1.48–2.12)	<0.001	1.41 (1.15–1.73)	<0.001	1.33 (1.09–1.63)	0.005
Stage < T2b	ref		ref		ref	
Stage ≥T2b	3.87 (2.53–5.93)	<0.001	1.63 (0.64–4.14)	0.304	1.70 (0.67–4.34)	0.264

**Fig. 5:**
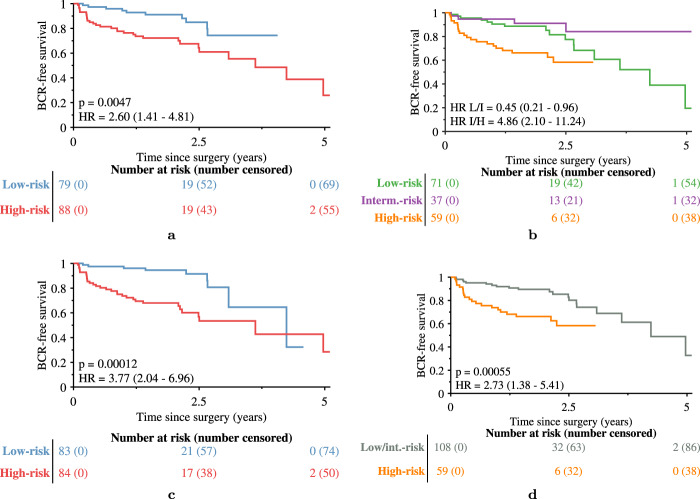
Kaplan-Meier BCR-free survival plots of the 167 patients of the validation set who had Decipher score information. Patients are stratified by (**a**) Histotyping, (**b**) Decipher risk groups, **c** Histotyping+, which incorporates Histotyping, pre-operative PSA, and Gleason grade group, and (**d**) Decipher risk groups with low and intermediate-risk as a single category.

For the *n* = 167 patients who had Decipher score information, to compare Decipher to Histotyping categorically, Decipher low-risk and intermediate-risk patients were grouped together as these groups did not have significantly different BCR-free survival (*p* = 0.14). Histotyping (*p* = 0.005, HR = 2.60, 95% CI: 1.41-4.81, c-index = 0.68, 95% CI: 0.59–0.74) performed slightly worse than Decipher (*p* < 0.001, HR=2.73, 95% CI: 1.38-5.41, c-index = 0.70, 95% CI: 0.61–0.78), as shown in Fig. 5. Histotyping+ surpassed Histotyping alone and Decipher (*p* < 0.001, HR = 3.77, 95% CI: 2.04–6.96, c-index = 0.75, 95% CI: 0.69–0.81) using five covariates selected by the model: Histotyping score, pre-operative PSA, and pathological Gleason grade groups 3, 4, and 5 (relative to 1). Though the 95% CIs of Histotyping+ and Decipher overlapped, Histotyping+ had the higher c-index in 81% of bootstrap iterations and the narrower 95% CI. In addition, Histotyping+ had a significantly higher c-index than Decipher in the bootstrap iterations (*p* < 0.001). In the validation set overall, Histotyping+ (c-index = 0.74) also outperformed a model using only pre-operative PSA and Gleason grade group (c-index = 0.72), as well as Gleason grade group (c-index = 0.69) and pre-operative PSA (c-index = 0.69) individually.

 Figure 1d shows an overlay of the corresponding Gleason grade group for each patient in the validation set within the UMAP embedding of the Histotyping features. As may be observed, higher Gleason grade group patients were more likely to be Histotyping high-risk. However, the concordance between Gleason grade groups and Histotyping-determined low- and high-risk patients appears to be weak to moderate at best (Pearson correlation coefficient = 0.37), in turn suggesting that Histotyping is capturing morphologic attributes at least partially complementary to Gleason grade.

## Discussion

Accurate post-surgery prostate cancer (PCa) biochemical recurrence (BCR) prognosis has substantial implications for patient care and healthcare utilization. While the STAMPEDE trial^13^ has demonstrated that adjuvant therapy can improve patient survival after radical prostatectomy (RP), not every patient will benefit from further treatment. It is possible that the use of a companion diagnostic to direct adjuvant therapy only to high-risk patients would have resulted in a larger benefit in the STAMPEDE trial^22^. However, there is a shortage of accurate prognostic tools for the post-RP setting. Based on current adjuvant therapy guidelines, the number needed to treat to prevent one death related to PCa is 10^23^. Existing BCR prognosis tools, nomograms, are driven by Gleason grading, which is limited by the power of human perception and has only moderate inter-reviewer agreement^3^. Accordingly, there has been an increasing awareness of the need for an objective and accurate BCR prognosis tool.

Genomic assays, such as the Decipher genomic test, have been validated for post-RP metastasis^16^ and BCR^20,21^ prognosis, but consume the tested tissue. While most RP specimens have an abundance of tissue, this requirement prohibits performing multiple tests on the same sample. In small tumors, the tumor tissue could be exhausted by repeated testing. In addition, molecular testing protocols are expensive and sophisticated, reducing their availability. These limitations make it infeasible to perform genomic testing on all the cancerous tissue in an RP specimen.

Recently, quantitative histomorphometric (QH) approaches have been proposed as a complement to molecular assays. While some work has linked QH features to PCa disease-free survival^24^, much of the literature has focused on automated Gleason grading and cancer detection^25–30^. In this work, we presented a QH-based assay, termed Histotyping, for post-RP PCa risk assessment. Histotyping uses an H&E slide and handcrafted features of gland morphology. Deep learning, in which a model maps images directly to labels with no other guidance, has been used in a range of digital pathology applications, including PCa grading^28–30^, microsatellite instability prediction^31^, and mutation prediction^32^. While deep learning approaches have produced promising results, their black-box nature means that model decisions are not always readily explainable or related to known pathological features. Histotyping leverages deep learning for lumen segmentation, from which handcrafted features are extracted. Since each feature in Histotyping is human interpretable, it is possible to scrutinize the model’s decisions and verify that Histotyping is properly quantifying tumor morphology.

Histotyping was prognostic of post-RP BCR-free survival in the validation cohort independent of common clinical markers, both as a continuous score and categorical low/high-risk division. This evaluation mirrors the design used to validate the Decipher genomic test^16^. Histotyping’s hazard ratio (HR) of 2.83 was similar to gold-standard nomograms (HR = 1.09–2.74)^33^. Histotyping+, incorporating Histotyping, Gleason grade group, and preoperative prostate-specific antigen level, had a higher concordance index than Histotyping alone and Decipher. The difference in performance between Histotyping+ and Decipher was significant, though the overlapping 95% confidence intervals of these models suggest that further study with a larger cohort may be necessary to increase confidence with regard to the degree of prognostic performance improvement. Histotyping added value in two cohorts which would be low-risk or intermediate-risk by current methods: patients with Gleason grade group 3 and those with negative surgical margins. This suggests Histotyping is able to identify patients at risk of BCR who would not be likely to be recommended for additional therapy under current schemes. Histotyping may be especially suitable for identifying high-risk patients with lower-risk clinical markers due to the lower hazard associated with additional adjuvant therapy versus de-intensifying therapy for clinically high-risk patients.

Of the six features selected for Histotyping, all but one were gland lumen shape descriptors. These findings suggest that, in addition to the overall appearance of the glands, the variation in lumen shape and architecture across a tumor is related to PCa aggressiveness. Specifically, a greater proportion of disk-shaped lumen within a tumor was associated with elevated BCR risk, and that the mixing of these disk-shaped lumens with elongated elliptical or crescent-shaped lumens further increased risk. The other features indicate that higher variation in gland density across the tumor carried a higher risk of BCR. The biological rationale for these features is further described in the supplementary material. These features were found to be stable across sites, despite the images being clearly affected by site-specific factors and batch effects.

These findings are consistent with other studies which have found that lumen shape, disorder, and texture features are useful for cancer detection^25,34^, grading^25–27^, and BCR prognosis^6,24^. However, previous work in this area has used a lower proportion of shape features, potentially because these studies did not consider inter-site feature instability. It is possible that studies using unstable features would have worse results on independent validation sets, as was found in Leo et al.^27^.

The performance of Histotyping suggests that morphology alone, from a single lesion on a single slide, has prognostic power comparable to gold-standard methods. The finding that gland lumen shape and architecture is correlated with BCR risk is not surprising, as the Gleason scoring method is based on similar features analyzed by a human pathologist^2^. In contrast to existing companion diagnostics, Histotyping requires only a routinely acquired diagnostic H*&*E slide, a whole-slide scanner, and a typical desktop computer.

A limitation to this study was that metastasis outcome was not available for these patients. While Decipher has been validated for BCR prognosis^20,21^, it was calibrated for metastasis^18^. A further limitation of Histotyping, shared by all existing PCa companion diagnostics, is that it has not been validated for treatment response prediction. Future work may include comparing Decipher and Histotyping in metastasis prognosis and in biopsy specimens where tissue for pathological and molecular analysis is more limited.

In addition, Histotyping relies on a pathologist for the selection of a slide and tumor region containing the most aggressive cancer, though this could be automated in future work and the results of the annotation modification experiment suggest that Histotyping is relatively robust to inter-reader disagreement in tumor boundary delineation. Directly testing the effect of inter-reviewer variation in slide and lesion selection and attempting to automate this process could be an avenue for future work. A single diagnostic slide was used here due to the prohibitive expense of locating and scanning all patient slides. While Histotyping examines only a sample of the overall tumor, far more tissue is interrogated than in molecular tests performed on tissue cores, which also rely on manual identification.

Though the multi-site validation included a variety of scanners, the effect of different scanners was not explicitly examined beyond the feature stability filtering used to mitigate such effects, a method shown to be effective in previous work^27,35,36^. In a related point, one-third of the training set (70 of 214 patients) was composed of patients from the University of Pennsylvania (UPenn). While these patients were collected separately and scanned on a different scanner compared to the UPenn patients in the validation set, there was some institutional overlap between the training and validation sets, and the Decipher validation set was made up almost entirely of UPenn patients. While it was possible for this to result in an over-optimistic estimate of model performance, this concern is somewhat mitigated by Histotyping performing better on the NewYork Presbyterian Hospital/Weill Cornell Medical Center (HR = 4.22) and The Cancer Genome Atlas (HR = 10.50) cohorts than on the UPenn cohort (HR = 2.34). Finally, Histotyping was not significantly prognostic in some subsets, such as the University of Turku cohort and in Gleason grade groups other than 3, though it achieved a hazard ratio >1 and *p* < 0.10 in grade groups 1, 2, and 4.

In this work, we have demonstrated an automated method that can stratify patients by BCR risk using a single H*&*E slide with performance similar to that of the Decipher molecular companion diagnostic.

## Methods

### Dataset description

**Fig. 6:**
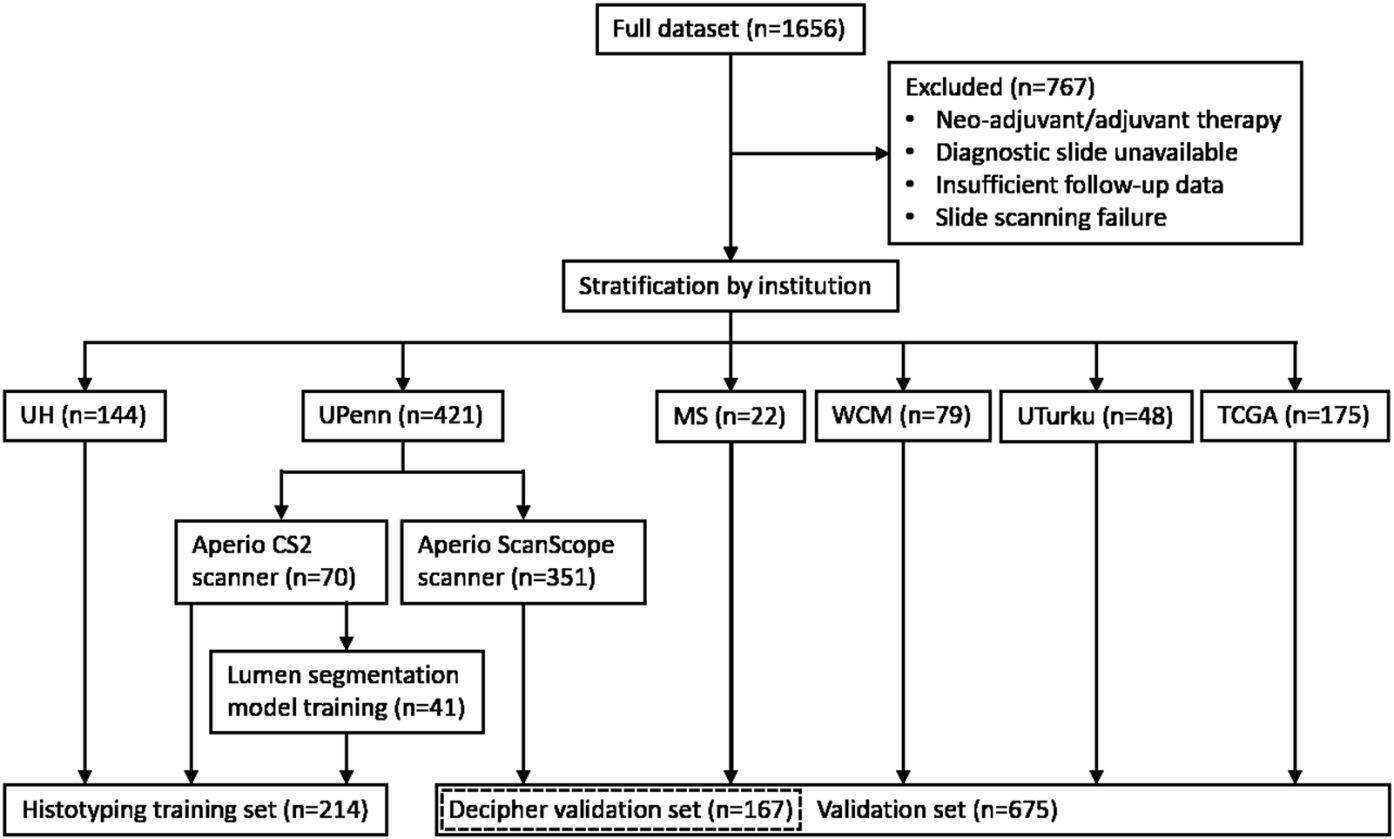
CONSORT diagram of the allocation of patients in this study.

This study used *n* = 889 patients (Table 3) from six sources: University of Pennsylvania (UPenn), University Hospitals Cleveland Medical Center (UH), NewYork-Presbyterian Hospital/Weill Cornell Medical Center (WCM), University of Turku (UTurku), The Cancer Genome Atlas (TCGA)^37^, and the Icahn School of Medicine at Mount Sinai (MS). Patients were digitized on a variety of whole-slide scanners (Supplementary Table 1). TCGA patients with discrepancies in outcome information were excluded^37^. A training set was selected to include patients from multiple institutions totaling approximately a quarter of the dataset and contained *n* = 70 UPenn patients and *n* = 144 UH patients. The validation set consisted of the remaining *n* = 675 patients from five sites (UPenn, WCM, UTurku, TCGA, MS), with no training set patients included in the validation set. UPenn patients were split between training and validation sets based on the scanner used. This division is shown visually in Fig. 6. Data collection was approved by institutional review boards at each institution and conducted in accordance with U.S. Common Rule guidelines. Specimen Gleason grades were assigned by a genitourinary specialist pathologist for all patients, with the potential exception of TCGA patients, where particulars of the grading pathologist were not available. The requirement for written consent from patients was waived due to the retrospective nature of the study.

**Table 3:** Clinical data for the 889 patients used in this study.

	Training set		Validation set				
Variable	UPenn	UH	UPenn	TCGA	WCM	UTurku	MS
Patients	70	144	351	175	79	48	22
Race, No. (%)							
Caucasian-American	63 (90.0)	91 (63.2)	232 (66.1)	70 (40.0)	0 (0.0)	0 (0.0)	17 (77.3)
African-American	7 (10.0)	35 (24.3)	111 (31.6)	3 (1.7)	79 (100.0)	0 (0.0)	2 (9.1)
Other	0 (0.0)	5 (3.5)	8 (2.3)	1 (0.6)	0 (0.0)	48 (100.0)	1 (4.5)
Unknown	0 (0.0)	13 (9.0)	0 (0.0)	101 (57.7)	0 (0.0)	0 (0.0)	2 (9.1)
Patient age, years (median [Q1, Q3])	59 (55, 65)	61 (57, 64)	61 (56, 66)	60 (55, 65)	61 (56, 66)	65 (61, 68)	62 (59, 67)
Unknown	0	129	0	0	0	24	0
pT stage, No. (%)							
2	36 (51.4)	9 (6.3)	163 (46.4)	90 (51.4)	63 (79.7)	28 (58.3)	14 (63.6)
3 (substaging unavailable)	0 (0.0)	2 (1.4)	1 (0.3)	0 (0.0)	0 (0.0)	3 (6.3)	0 (0.0)
3a	23 (32.9)	5 (3.5)	139 (39.6)	53 (30.3)	11 (13.9)	12 (25.0)	4 (18.2)
3b	11 (15.7)	2 (1.4)	48 (13.7)	27 (15.4)	5 (6.3)	3 (6.3)	4 (18.2)
4	0 (0.0)	0 (0.0)	0 (0.0)	2 (1.1)	0 (0.0)	0 (0.0)	0 (0.0)
Unknown	0 (0.0)	126 (87.5)	0 (0.0)	3 (1.7)	0 (0.0)	1 (2.1)	0 (0.0)
N stage, No. (%)							
0	70 (100.0)	11 (7.6)	345 (98.3)	126 (72.0)	78 (98.7)	35 (72.9)	21 (95.5)
1	0 (0.0)	0 (0.0)	3 (0.9)	24 (13.7)	1 (1.3)	1 (2.1)	1 (4.5)
Unknown	0 (0.0)	133 (92.4)	3 (0.9)	25 (14.3)	0 (0.0)	12 (25.0)	0 (0.0)
Pre-operative PSA, ng/mL (median [Q1, Q3])	8 (5, 11)	6 (5, 8)	6 (5, 9)	6 (5, 9)	6 (4, 8)	8 (6, 10)	8 (5, 13)
Unknown	0	66	4	5	3	1	0
RP Grade Group, No. (%)							
1	8 (11.4)	33 (22.9)	74 (21.1)	20 (11.4)	17 (21.5)	9 (18.8)	1 (4.5)
2	38 (54.3)	80 (55.6)	163 (46.4)	73 (41.7)	40 (50.6)	17 (35.4)	13 (59.1)
3	16 (22.9)	11 (7.6)	68 (19.4)	43 (24.6)	15 (19.0)	8 (16.7)	4 (18.2)
4	4 (5.7)	2 (1.4)	22 (6.3)	23 (13.1)	1 (1.3)	4 (8.3)	0 (0.0)
5	4 (5.7)	4 (2.8)	22 (6.3)	16 (9.1)	6 (7.6)	9 (18.8)	4 (18.2)
Unknown	0 (0.0)	14 (9.7)	2 (0.6)	0 (0.0)	0 (0.0)	1 (2.1)	0 (0.0)
Positive surgical margins, No. (%)	30 (42.9)	9 (6.3)	208 (59.3)	29 (16.6)	8 (10.1)	7 (14.6)	1 (4.5)
Unknown	0 (0.0)	129 (89.6)	1 (0.3)	16 (9.1)	0 (0.0)	1 (2.1)	0 (0.0)
Follow-up of censored patients, years (median [Q1, Q3])	2 (1, 4)	7 (5, 9)	2 (2, 5)	1 (1, 3)	2 (0, 4)	2 (2, 4)	0 (0, 1)
Patients with BCR, No. (%)	35 (50.0)	45 (31.3)	115 (32.8)	7 (4.0)	10 (12.7)	11 (22.9)	5 (22.7)
Unknown	0 (0.0)	0 (0.0)	0 (0.0)	0 (0.0)	0 (0.0)	0 (0.0)	0 (0.0)

Inclusion criteria were a successfully digitized diagnostic slide, post-RP PSA test results for at least 30 days post-surgery, and no neo-adjuvant or adjuvant therapy. Patients were labeled BCR at the date of second PSA serum tests >0.2 ng/mL or censored at the date of the last available PSA test.

A subset of the validation set consisting of 145 UPenn and 22 MS patients had Decipher genomic classifier^18^ results available and were used to compare Histotyping to Decipher. These UPenn patients constituted a consecutive cohort operated on by a single surgeon.

The highest grade (for UTurku patients) or diagnostic (for all other sources) slide of each patient was digitized in a whole-slide scanner. Slide and tumor nodule used were determined by a genitourinary pathologist. A single representative cancerous region with at least 10 glands, selected to include the highest grade cancer on the slide, was annotated on each digital image. This mirrors the manual selection of a representative tissue block for molecular companion diagnostic tests^38^. One pathologist (X.F.) annotated the images from UTurku, a second pathologist (R.E.) annotated all other images. Training set images also had a representative non-cancerous region annotated for the feature stability filtering step of model training.

### Histotyping construction

**Fig. 7:**
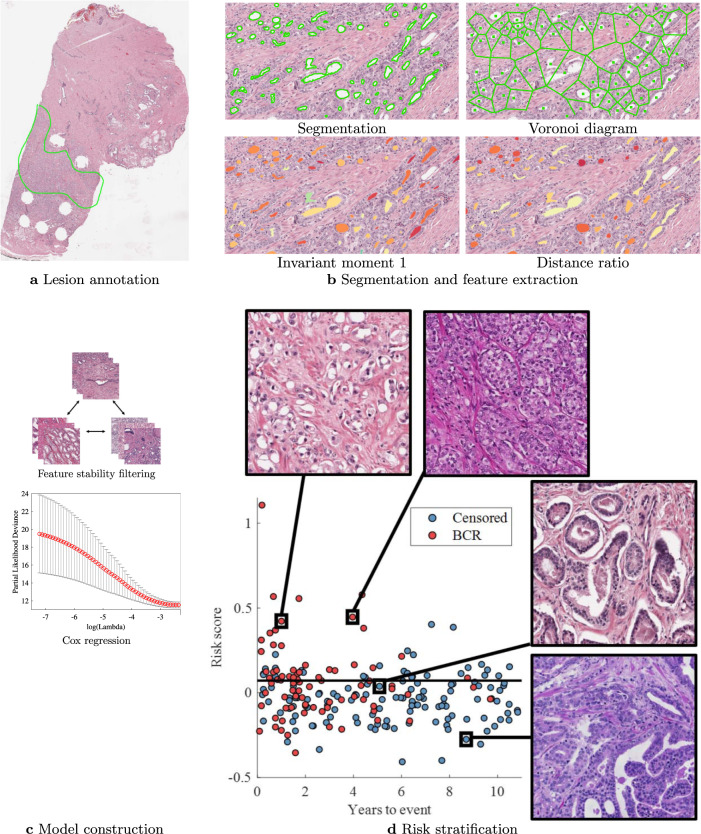
Diagram of the Histotyping development process. **a** Pathologist annotation of a representative tumor region on a whole-slide image. **b** Result of automated lumen segmentation and feature visualizations from a region of interest in the pathologist-annotated tumor region. Shown is a Voronoi diagram, constructed by edges that are equidistant from adjacent glands, the first invariant moment, which is equivalent to moment of inertia, and the distance ratio, which is the ratio of a gland’s average radius to its maximum radius. In the invariant moment 1 and distance ratio figures, glands are shaded from yellow to red according to their feature values. **c** Steps of model training, where features are filtered for stability using the three cohorts of the training set. A Cox regression model was then fitted using 10-fold elastic-net regularization. **d** Results of model training. Each patient of the *n* = 214 training set is represented as a dot in the scatter plot, with patients who had BCR as red dots and censored patients as blue dots. Dots are located on the *x*-axis at their time of BCR or time of last PSA test and on the *y*-axis at their Histotyping risk score from the Cox regression model. The stratification threshold identified on the training set is shown as a horizontal black line and was then used to classify patients as low-risk or high-risk. Regions of interest from patients at various risk scores are shown in boxes.

The Histotyping design workflow is shown in Fig. 7. Lumen were first segmented to enable feature extraction. This segmentation was performed by a UNet-inspired^39^ deep learning model. Images were resized to 1 micron-per-pixel (×10 magnification) resolution for this step. The model was trained on 41 1000 × 1000 pixel regions cropped from 37 training slides annotated for a total of 4927 gland lumens. On the four regions held out for testing, the model yielded a per-pixel true positive rate of 0.94, a true negative rate of 0.97, and an F1 score of 0.90. Segmentation was performed on all 889 images, and results were visually examined, with segmentations found to be sufficiently accurate for feature extraction.

A total of 242 features were extracted from the annotated tumor region on each slide, of which a subset of six was used in Histotyping. 216 features were descriptors of morphology and architecture extracted from lumen segmentation. 26 Haralick texture features were extracted from the tumor region, disregarding the segmentations. These features were selected based on their performance in PCa grading^27^ and BCR prognosis^6^.

All 242 features were subjected to filtering for stability across sites using the method of Leo et al.^34^ on the training set. Features that passed stability filtering were used to train a Cox regression model via 10-fold elastic-net regularization (*α* = 0.5)^40^. In this process, the set of features and their weights in the model were optimized by minimizing a penalty term consisting of both the model error in the training set and the sum of feature weights. This approach performs feature selection, by forcing the weights of some features to zero, and model fitting, by selecting the weights associated with the lowest error on the training set. Features were normalized using the training set to have a mean of 0 and standard deviation of 1 so that HRs would be comparable across features. The final model, containing six features, was then applied to all slides to obtain a risk score for each patient. A threshold was learned on the training set to stratify patients as low-risk or high-risk. The supplemental material includes details of the segmentation procedure, model framework, and extracted features.

### Evaluating reproducibility of Histotyping

UMAP embedding^41^ was performed on the validation set to assess the inter-site variation between images prior to Histotyping analysis and to verify that Histotyping features were resilient to batch effects across multiple sites. Such sources of pre-analytic variation can arise from differences in specimen preparation and scanning between laboratories, are correlated with the site, and have been shown to degrade the performance of digital pathology analysis algorithms^34,42,43^. UMAP was used to reduce the feature space to two dimensions for evaluating the clustering between slides from different laboratories. If distinct clusters emerged in the UMAP space and those clusters corresponded to slides from a specific site, that would reflect the presence of site-specific attributes or batch effects. On the other hand, if the images from all sites were more homogeneously distributed in the UMAP space, it would suggest that the original set of features was resilient to the batch effects.

The UMAP embedding was performed on both the (1) original set of images and then (2) the set of Histotyping features from the same set of images. This was done to test the hypothesis that, though the input images exhibited site-specific effects and would cluster in the UMAP space, the features extracted for Histotyping were stable across sites. A digital pathology analysis tool, HistoQC^42^, was used to extract 29 quantitative metrics describing the brightness, contrast, color distribution, and stain intensities from the validation set images. A full list of metrics is available in the supplemental material. The embedding was repeated on the same set of images, but this time on the six features comprising Histotyping.

### Simulating the effect of inter-reviewer variability in tumor annotation on Histotyping

To test the robustness of Histotyping to simulated inter-reviewer variation in tumor annotation, Histotyping analysis was rerun on each validation set image by iteratively perturbing and eroding the original tumor annotation. Since reader disagreement on the cancerous region was most likely to occur at the edges of the tumor, where cancerous glands begin to mix with non-cancerous ones, the outer layer of glands was removed from each annotation and the resulting Histotyping score was recalculated. This process was repeated 10 times on every validation set annotation, at which point a layer 10 glands thick had been removed from the original annotation (see Fig. 2). This simulated the effect of variation in pathologist annotation of tumor extent. The c-index of Histotyping and fraction of patients whose Histotyping risk category changed at each boundary layer removal was measured. If Histotyping was highly sensitive to the precise extent and annotation of the cancerous region, a large number of patients changing risk categories and steep drop-off in c-index would be observed.

### Histotyping performance evaluation

The performance of Histotyping was evaluated in the validation set using the separation in BCR-free survival time between the low-risk and high-risk groups by logrank *p*-value, hazard ratio, and Harrell’s c-index. Model independence was evaluated in a Cox proportional hazards model with Histotyping risk score, Gleason grade group, margin positivity, pathological tumor stage, and preoperative PSA. Clinically stratified cohorts were analyzed separately to determine if Histotyping added value. Two such cohorts (Gleason grade group 3, margin negative) are discussed here, with further results in the supplemental material.

### Comparison of Histotyping and decipher

Histotyping was compared to Decipher for BCR prognosis in the 167 patients of the validation set who had Decipher score information. Decipher scores were calculated based on the predefined 22-marker Decipher classifier^18^. The Decipher score is a score between 0 and 1, with lower scores indicating a lower risk of metastasis. Decipher categorizes patients as high-risk (Decipher score >0.60), intermediate-risk (0.45–0.60), or low-risk (<0.45).

In addition, a second elastic-net Cox model was constructed on the training set using the continuous Histotyping score, preoperative PSA level, and Gleason grade group to create the Histotyping+ model. These covariates were chosen as they were available in *n* = 148 training set patients, more than for any other set of covariates. Histotyping+ was compared to Decipher by c-index in the *n* = 167 patients of the Decipher validation set in absolute terms and in 1000 iterations of bootstrapping. The 95% CI of c-index was computed from these bootstrap iterations and a two-tailed t-test was used to test for a significant difference in the distributions. For low/high-risk stratification, a new decision threshold was chosen using the training set in the same process as for Histotyping.

### Reporting summary

Further information on research design is available in the Nature Research Reporting Summary linked to this article.

## Supplementary information

Supplementary Information

Reporting Summary

## Data Availability

The data generated and analyzed during this study are described in the following data record: 10.6084/m9.figshare.14226278^44^. The following files are openly available as part of this data record: the Histotyping scores of each patient in the training set (*n* = 214) in the file “training_set_HT_scores.xlsx”; the Histotyping scores of each validation set patients during successive boundary layer removals in the file “boundary_layer_data.xlsx’; image metrics, Histotyping scores, Histotyping+ scores, and UMAP components for each patient in the validation set in the file “HT_UMAP_supporting_data.xlsx”; ground truth masks and segmentation results on lumen segmentation model validation set images in the folder “gland_segmentations.zip”. The patient clinical data are contained in the Excel spreadsheet “patient_clinical_info.xlsx”. These data are not publicly available for the following reason: material transfer agreements from source hospitals do not allow public sharing of patient information. However, the data can be made available upon reasonable request to the corresponding author.

## Code availability

This study made use of publicly available code^41,45,46^, with in-house developed feature extraction code available at https://github.com/pjl54/matlab-feature-extraction.
