# Supplementary material for: Computer extracted gland features from H&E predicts prostate cancer recurrence comparably to a genomic companion diagnostic test: a large multi-site study
Source: NPJ Precis Oncol. 2021 May 3;5:35. doi: 10.1038/s41698-021-00174-3 (PMC8093226; doi:10.1038/s41698-021-00174-3)
Supplement: Supplementary file 1 — Supplementary Information [file 41698_2021_174_MOESM1_ESM.pdf]

Supplementary information for:

Computer extracted gland features from H&E predicts prostate cancer recurrence comparably to a genomic companion diagnostic test: a large multi-site study

---

---

1. Gland lumen segmentation

The steps for training the gland lumen segmentation model were as follows:

1. A 2000 x 2000 pixel region of interest (ROIs) at 0.5 microns-per-pixel (20X magnification) was selected from the tumor regions of 29 slides of the training set. ROIs were selected to provide a representative range of tissue morphology and were therefore drawn from slides varying in cancer grade and staining presentation.
2. ROIs were manually annotated for gland lumen by a resident pathologist (N.J.). Annotations were performed using QuPath v0.12 [1] on a traditional desktop system with a mouse. The annotations were then manually refined to ensure maximum fidelity to the lumen boundary by correcting inaccuracies stemming from the coarseness of the initial annotations. A total of 4927 lumens were annotated in this manner.
3. ROIs were resized to 1 micron-per-pixel resolution.
4. A UNet-inspired [2] deep learning model was trained using these 29 ROIs. This network architecture consisted of approximately 2 million parameters, with 4 max-pooling layers along the networks depth.
5. Model performance was qualitatively evaluated on whole slide images from the Histotyping training set to identify images with poor segmentation performance. To improve model performance, an additional 12 1000 x 1000 micron ROIs exhibiting morphology the model performed poorly on were selected, annotated, and added to the model training set. These additional 12 ROIs brought the total number of ROIs used for lumen segmentation model development to 41, drawn from 37 different slides.
6. The UNet-inspired model was then retrained on the total set of 41 annotated ROIs. Four of these ROIs, shown in Figure 1, were used for model testing. The testing ROIs came from slides not used in the 37 ROI training set.

The model weights used in this study were derived from the epoch which minimized the loss function value on the four validation images. The optimal loss was reached after 290 epochs, which coincided with 103.5 minutes using a Nvidia Titan Xp GPU. Further training for a total of 1000 epochs did not produce a smaller validation loss value.

In training of each UNet model, data was augmented through vertical and horizontal flipping, resizing and cropping, rotation, and brightness, contrast, saturation, and hue transforms.

Following deep learning segmentation, results were further processed to remove artifacts by the following steps:

1. Segmented objects too small to be legitimate lumen (area < 4 square microns) were removed. The area threshold was set to be just below the smallest lumen annotated by the pathologist.
2. Segmented objects which, when dilated by 1 pixel, had a border containing more than 5% white pixels were removed. This was done to eliminate erroneous segmentations which had a border touching white pixels, since no legitimate lumen could have a boundary defined by white pixels.
3. Holes in segmentation results were filled.

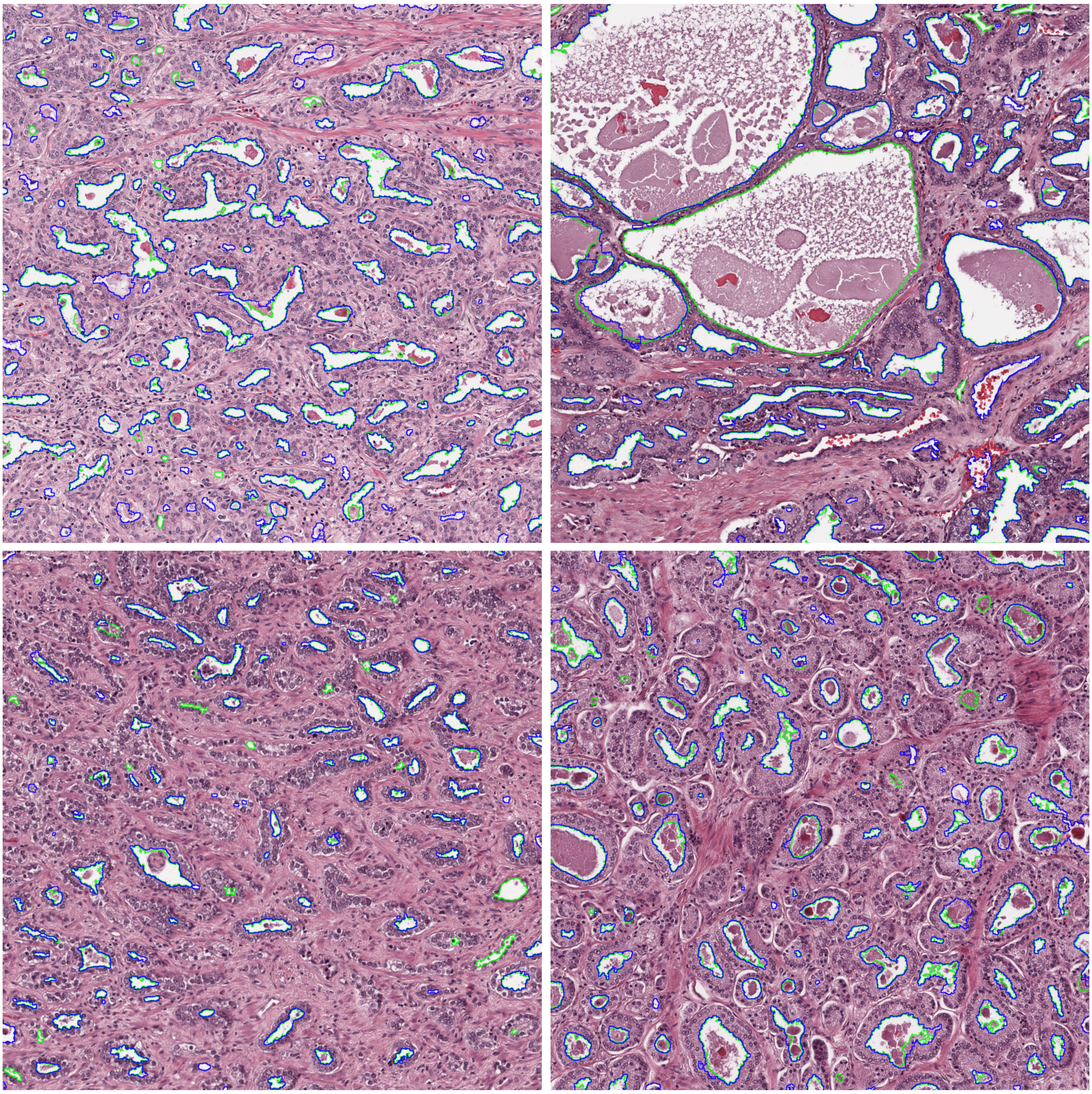

Supplementary Figure 1: Ground truth annotations (green) and segmentation results (blue) for the four held-out test images. The model achieved a pixel-wise true positive rate of 0.97 and a true negative rate of 0.97 on these four images.

2. Classifying a new patient

The steps to perform Histotyping classification on a new image, such as an image in one of the validation cohorts, are as follows:

1. A pathologist annotates a representative tumor region on the digital image.
2. Gland lumens from within the annotated tumor boundary are segmented as described in section 1.
3. 216 gland lumen features are extracted from the segmentation results. In addition, 26 Haralick texture features are extracted from the entire annotated region. This results in a 242 element feature vector.
4. All features are normalized by subtracting the 242 element vector of the training set feature mean values and element-wise division by the 242 element training set feature standard deviation values.
5. The normalized feature vector is multiplied by a vector of β values from the trained model. All but 6 elements of the β vector, corresponding to the features included in the model, are zero. The sum of the products of the normalized feature vectors and their corresponding β values is the Histotyping risk score
6. The risk score threshold identified on the training set is applied to the risk score from the previous step to categorize the patient as Histotyping low-risk or high-risk according to whether the image risk score is less than or greater than or equal to the threshold value.

3. HistoQC image metrics

For UMap embedding based on image metrics, HistoQC software downloaded from https://github.com/choosehappy/HistoQC was used to extract the following 29 quantitative metrics of image appearance from the images of the validation set: bright, dark, template1_MSE_hist, template2_MSE_hist, template3_MSE_hist, template4_MSE_hist, tenenGrad_-contrast, michelson_contrast, rms_contrast, grayscale_brightness, grayscale_brightness_std, chan1_brightness, chan1_-brightness_std, chan2_brightness, chan2_brightness_std, chan3_brightness, chan3_brightness_std, chan1_brightness_-YUV, chan1_brightness_std_YUV, chan2_brightness_YUV, chan2_brightness_std_YUV, chan3_brightness_YUV, chan3_-brightness_std_YUV, deconv_c0_mean, deconv_c0_std, deconv_c1_mean, deconv_c1_std, deconv_c2_mean, deconv_-c2_std.

4. Stability filtering

Since patients in the study dataset originated from many institutions with different protocols and equipment, there was variability in specimen preservation, fixation, sectioning, and staining as well as slide digitization hardware. These sources of pre-analytic variation affect the final appearance of the slide images and thereby the features extracted from these images.

Therefore, features highly susceptible to site-specific factors were removed to improve model performance [3]. For this step, the training set was divided into sub-cohorts. The UPenn set contained two sub-cohorts, containing 30 and 40 patients, which were collected at different times and were qualitatively different. The UH patients were divied into two sub-cohorts according to scanner used to digitize specimens. This analysis was restricted to the non-cancerous regions containing at least 10 glands to eliminate the confounding effect of tumor morphology on stability calculations. Note that this was the sole step of this study employing non-cancerous regions. These restrictions caused the three sub-cohorts used in this step to contain 36, 38, 73, and 20 patients.

Using the method of Leo et al. [4], three quarters of the patients in each sub-cohort were randomly selected and features were evaluated with the Wilcoxon rank sum test for significant difference between each pair of cohorts. This sub-sampling and comparison was repeated 1000 times. Features significantly different in more than 10% of iterations were discarded.

Supplementary Table 1: Number of patients, median BCR and follow-up times, and scanner hardware for each site.

Site	n (n BCR)	Med. BCR (censor) year	Scanner hardware (magnification)
UPenn (Training)	70 (35)	1.7 (1.8)	Aperio CS2 (20X)
UPenn (Validation)	351 (114)	1.7 (2.3)	Aperio ScanScope (40X)
UH	144 (36)	2.0 (6.6)	27 on Aperio SCN400 (40X)<br>36 on Aperio SCN400 (20X)<br>81 on Zeiss Axio Scan.Z1 (40X)
TCGA	175 (7)	0.5 (1.5)	Various Aperio scanners, models unknown (40X)
WCM	79 (10)	1.0 (2.9)	Aperio AT2 (40X)
UTurku	48 (13)	2.0 (2.5)	15 on Aperio SCN400 (40X)<br>33 on Hamamatsu Nanozoomer S60 (20X)
MS	22 (6)	0.2 (0.4)	Pannoramic 250 FLASH II (40X)
**Total**	889 (228)	1.7 (2.2)	

5. Thresholding risk scores

To find the optimal stratifying threshold of low- and high-risk patients, each midpoint of the risk scores of consecutive training set patients was considered. First, thresholds which yielded a group smaller than one-third of the training set or a logrank p-value >0.05 were discarded. Next, the set of thresholds which yielded the maximum absolute difference in median survival time between the groups was identified. From among identified thresholds, the one with the largest hazard ratio was selected and applied to the training and validation sets to create the Histotyping risk groups.

6. Concordance of Histotyping and Decipher

To investigate the concordance of Histotyping risk categories with Decipher risk categories, a set of N=66 patients from the Cleveland Clinic were analyzed alongside the patients of the validation set who had Decipher score information. BCR outcome information was not available for Cleveland Clinic patients, which is why those patients were not included in the validation set. The distribution of Histotyping risk scores for patients in each Decipher risk Category are shown in Figure 10.

7. Analysis of features used in Histotyping

The Histotyping risk assessment model for prostate cancer is based on six features of gland morphology extracted from automated segmentations of gland lumens. This supplementary material provides a description and visualization of each of these features. The features used in Histotyping broadly fell into three categories. These categories included features of (1) lumen shape diversity, (2) average lumen shape, (3) lumen density uniformity. The three features in the first category captured the variation in lumen shape across the tumor, with lower variation in lumen shape being associated with a higher risk of biochemical recurrence (BCR). The two features in the second category described typical lumen shape, with particular lumen shapes being associated with a higher risk of BCR. The final category had a single feature describing the variation in lumen density, with tumors with small regions of densely packed glands divided by large amounts of stroma being associated with increased BCR risk.

Features describing variation in lumen shape

In this study, lower variation in lumen shape was found to be associated with higher BCR risk following radical prostatectomy. This may be due to the tendency of poorly formed glands to have a smaller range of lumen shapes compared to well-formed glands [5], and a preponderance of poorly formed glands to be associated with worse outcomes. This finding is consistent with the criteria of the Gleason grading system in which pattern 3, a lower-risk morphology, includes subtypes with a range of lumen shapes, such as meandering and branching, or large and

atrophic, or ductal and fin-like. In contrast, the smaller gland lumen that are associated with Gleason pattern 4, seen in higher-risk tumors, tend to be smaller than those of pattern 3 [6]. Though pattern 4 glands themselves have a variety of shapes, the small size of the lumens limits the potential diversity of shapes and those that exist tend to be mostly circular.

Shape feature: Standard deviation of invariant moment 1

The invariant moments are a set of mathematical shape descriptors [7]. In particular, the first invariant moment is equivalent to the moment of inertia about the object's center. An object's moment of inertia refers to how much force would be needed to make it spin faster. So while a disk, with a low moment of inertia, would be easy to spin, it would require more force to start spinning a dumbbell of the same mass [8].

In the context of lumen shape, this means a low invariant moment 1 is associated with a disk-like shape while a high invariant moment 1 is associated with lumen that are elongated or have much of their area far from their center. As a result, the standard deviation of invariant moment 1 is low for a collection of disk-like objects, which have similar values for this feature, and high for a set of diverse shapes. So a tumor composed of mostly Gleason pattern 4 tissue with circular lumen will have a low standard deviation of invariant moment 1, reflecting concordance between the features used by Histotyping and the Gleason grading system.

Shape feature: 5th/95th percentile ratio of Fourier descriptor 6 & Shape feature: 5th/95th percentile ratio of Fourier descriptor 9

Fourier descriptors summarize the shape of an object as a series of numbers with the lower numbered descriptors describing overall shape (the low frequency information) and the higher numbered descriptors capturing the finer details of the lumen boundary [9, 10]. As a result, lumens with similar values for their Fourier descriptors will look similar. Figure 3 visualizes the Fourier descriptors by reconstructing the lumen segmentations with various numbers of descriptors. While the overall shape of the lumen, that of an ellipse, emerges from the first three descriptors, the finer details of the lumen boundary are produced by the higher-numbered descriptors. Formally, the higher-numbered Fourier descriptors describe the change in lumen boundary direction over very short distances while the lower-numbered descriptors measure changes in lumen boundary over long distances [9]. Mathematically, the Fourier descriptors of a gland lumen are calculated by performing the discrete Fourier transform on the coordinate pairs defining the boundary pixels of that lumen.

The ratio of the 5th to 95th percentile values of two Fourier descriptors were both negatively associated with BCR risk. This means a larger range of Fourier descriptor values, indicative of a larger range of lumen shapes, was correlated with lower risk of BCR. As described previously, Gleason pattern 3 tissue with produce a greater diversity of lumen shapes than pattern 4 tissue, providing a pathological rationale for the prognostic power of this feature.

Features describing average lumen shape

Shape feature: Median mean/maximum ratio of lumen radius

The ratio of the mean lumen radius to the maximum lumen radius is a measure of the regularity of the lumen boundary. A perfect circle has a mean/maximum radius ratio equal to 1, since every line from the circles center to a point on its boundary is the same length. On the other hand, a circle with a spike shaped protrusion would have a low mean/maximum ratio, as the radius at the protrusion would be much higher than the average of the radius at every other boundary point. A high average mean/maximum radius ratio was associated with a higher risk of BCR in Histotyping, as illustrated in Figure 6.

Shape feature: Mean Fourier descriptor 3

As described above, the Fourier descriptors are a way of distilling the overall shape of an object into a number, just as frequency is a single number describing a one dimensional function. A higher average value of Fourier descriptor 3 across the lumens of a tumor was associated with elevated BCR risk. The lumens with a higher Fourier descriptor 3 value, examples of which are shown in Figure 7, may be associated with a more aggressive cancer phenotype.

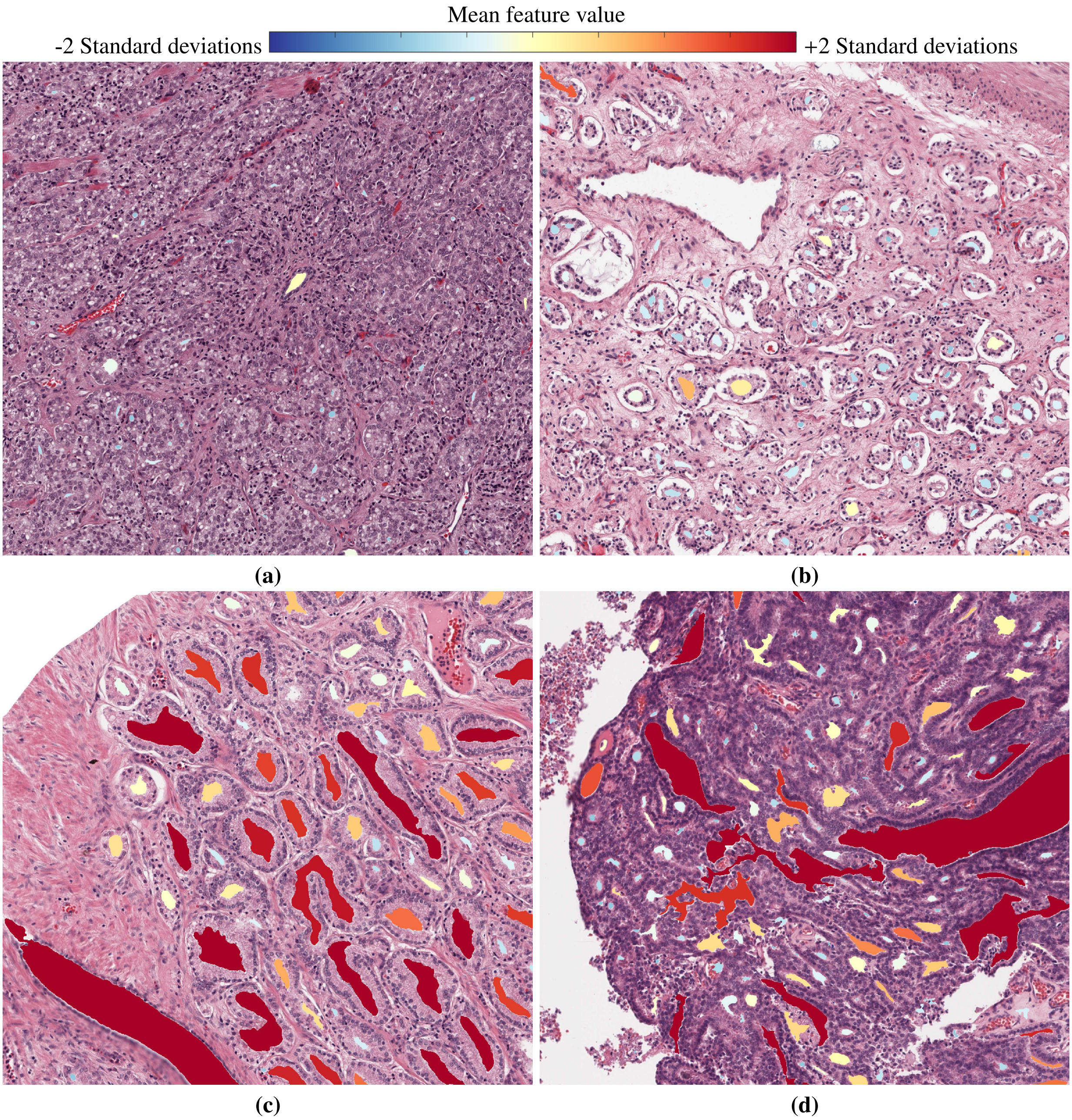

Supplementary Figure 2: Visualization of the invariant moment 1 feature on automatically segmented gland lumens with (a)(b) low and (c)(d) high values for the standard deviation of invariant moment 1. Lumens are colored according to their relative invariant moment 1 value, from low (blue) to high (red). A low standard deviation of invariant moment 1, as seen in (a) and (b) would contribute to Histotyping identifying patients as high-risk. Higher variance in invariant moment 1, visually evident from the variety of colors in (c) and (d), is associated with a greater number of well-formed glands with diverse lumen shapes.

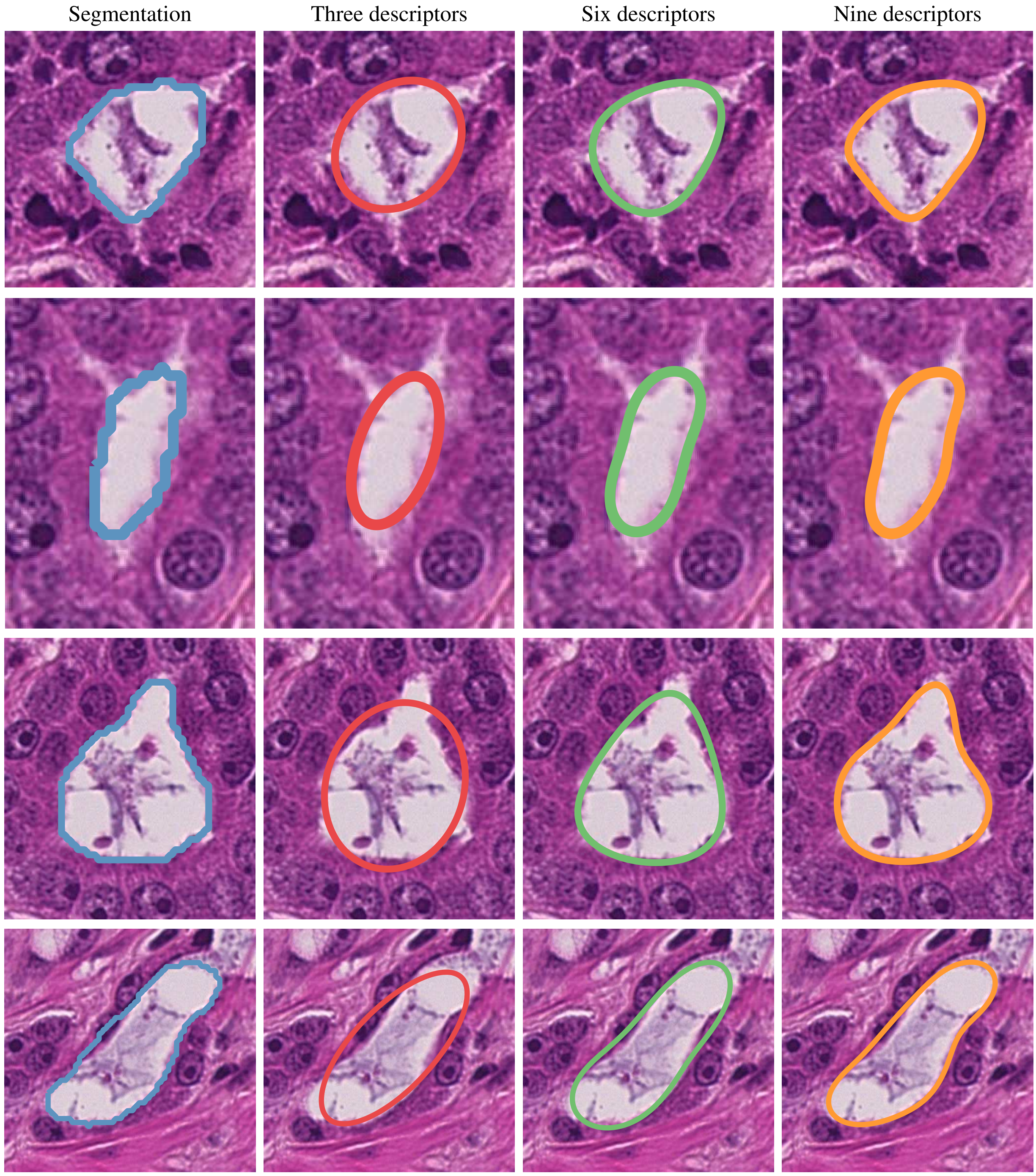

Supplementary Figure 3: Automated gland lumen segmentations are shown in blue in the leftmost column and reconstructed using various numbers of Fourier descriptors in the following columns. The lower numbered Fourier descriptors encode information about the lumen's overall shape, causing every lumen to be a circle when reconstructed with three Fourier descriptors. Higher numbered descriptors capture the smaller details of the lumen boundary, as seen in the reconstructions with nine Fourier descriptors which more closely resemble the original segmentations.

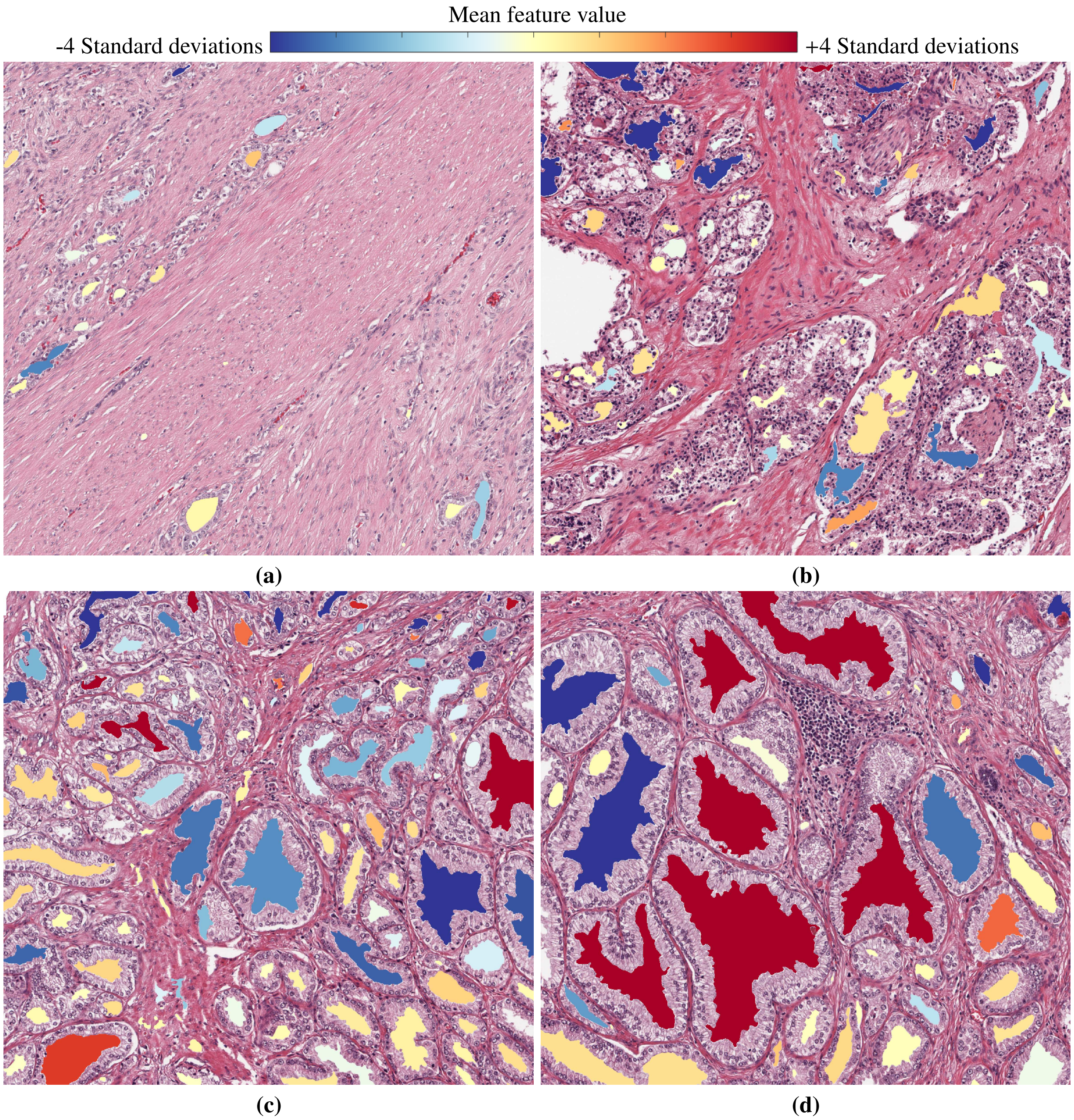

Supplementary Figure 4: Visualization of a lumen shape feature, Fourier descriptor 6, on automatically segmented gland lumens with (a)(b) low and (c)(d) high values for the 5th percentile / 95th percentile ratio of Fourier descriptor 6. Lumens are colored according to the their Fourier descriptor 6 value, from low (blue) to high (red). A low 5th percentile / 95th percentile ratio of Fourier descriptor 6, as seen in (a) and (b) would contribute to Histotyping identifying patients as high-risk. A larger range of Fourier descriptor 6 values, visually evident from the wider range of colors in (c) and (d), is associated with a greater number of well-formed glands with diverse lumen shapes.

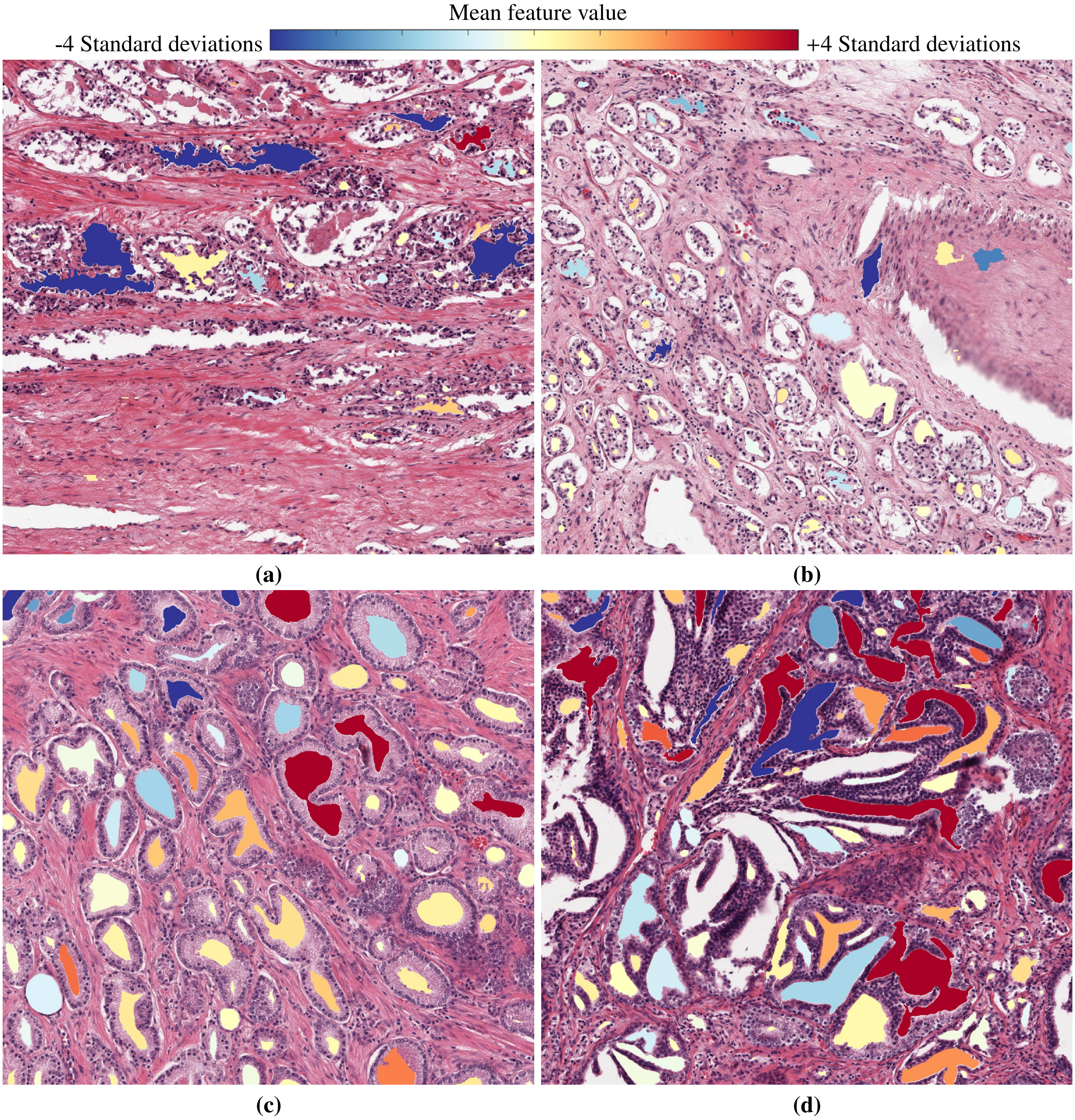

Supplementary Figure 5: Visualization of a lumen shape feature, Fourier descriptor 9, on automatically segmented gland lumens with (a)(b) low and (c)(d) high values for the 5th percentile / 95th percentile ratio of Fourier descriptor 9. Lumens are colored according to their Fourier descriptor 9 value, from low (blue) to high (red). A low 5th percentile / 95th percentile ratio of Fourier descriptor 9, as seen in (a) and (b) would contribute to Histotyping identifying patients as high-risk. A larger range of Fourier descriptor 9 values, visually evident from the greater variety of colors in (c) and (d), is associated with a greater number of well-formed glands with diverse lumen shapes.

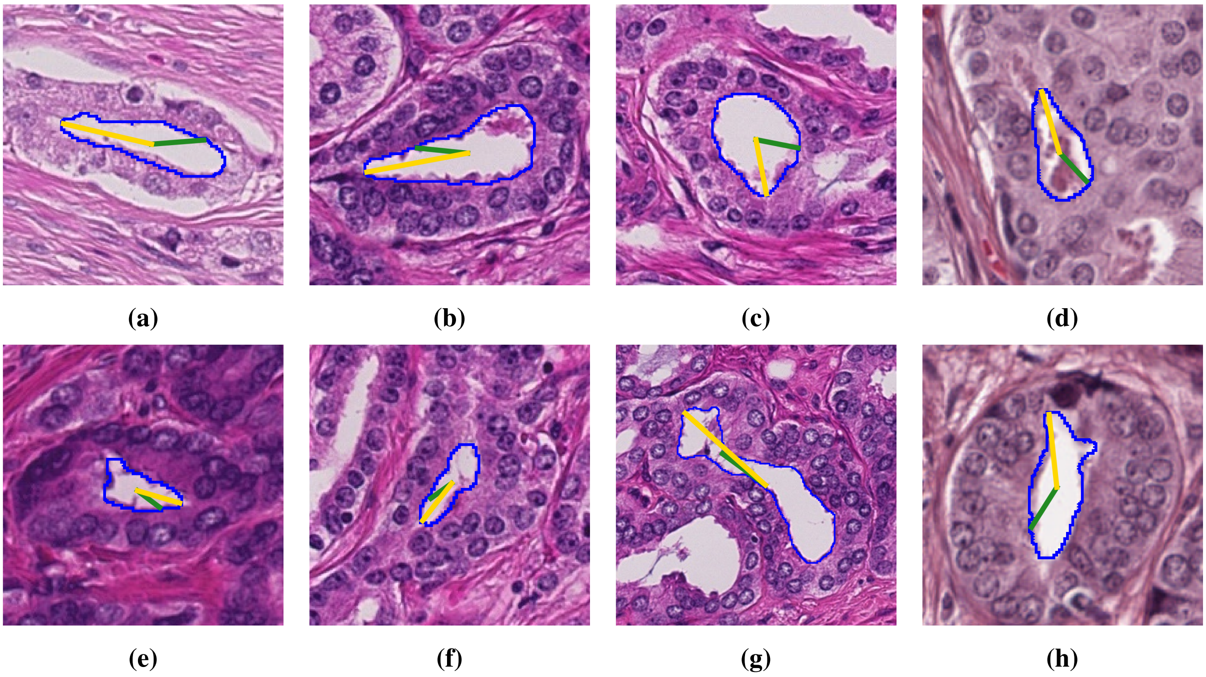

Supplementary Figure 6: Visualization of the mean/maximum lumen radius feature on automatically segmented gland lumens, whose boundaries are shown in blue. The maximum radius is denoted by the yellow line, while the mean radius is denoted by the green line. Shown are examples of (a-d) low mean/maximum ratio lumens and (e-h) high mean/maximum ratio lumens. A high mean/maximum ratio, as seen in (e-h) would contribute to Histotyping identifying patients as high-risk.

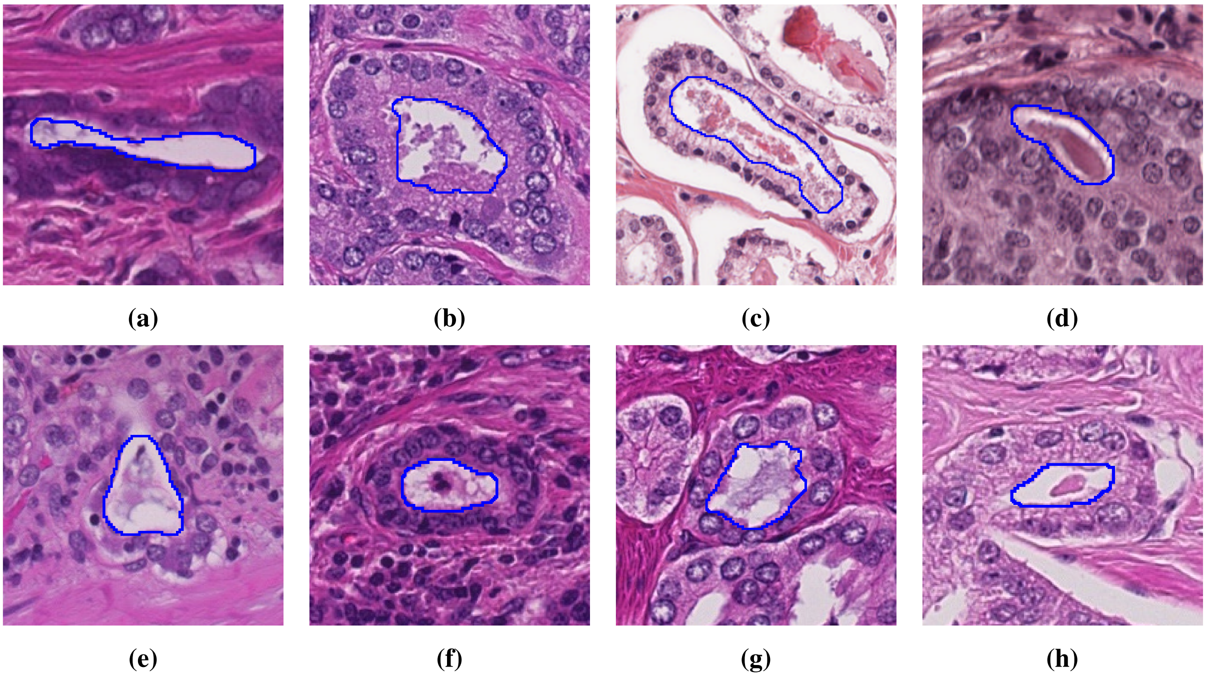

Supplementary Figure 7: Visualization of Fourier descriptor 3 on automatically segmented gland lumens, whose boundaries are shown in blue. Shown are examples of (a-d) low Fourier descriptor 3 lumens and (e-h) high Fourier descriptor 3 lumens. A high average Fourier descriptor 3 value, as seen in (e-h) would contribute to Histotyping identifying patients as high-risk.

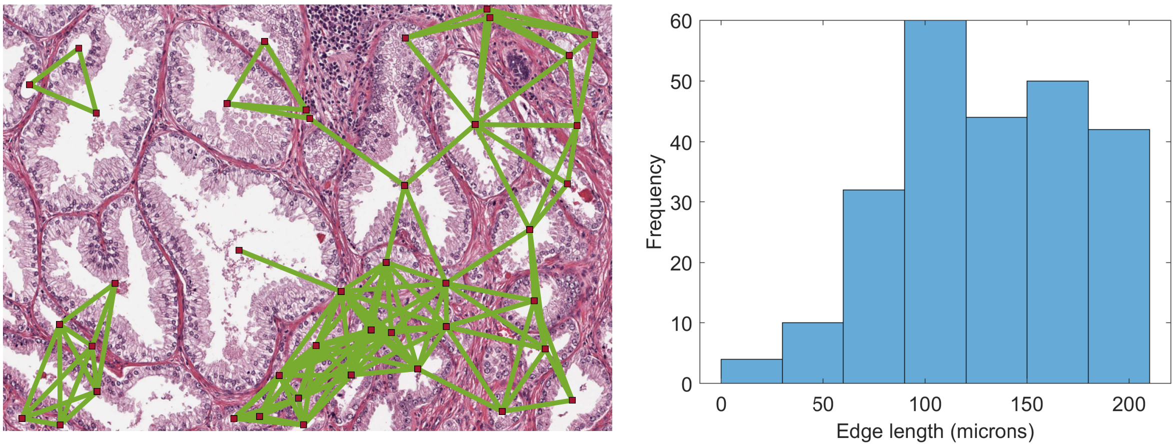

(a) **(b)**

Supplementary Figure 8: (a) An example sub-graph, which captures gland lumen arrangement by connecting nearby lumens with edges, indicated by green lines. (b) The distribution of edge lengths in (a). The kurtosis of this particular distribution is 2.32, very close to the average kurtosis value in the Histotyping training set overall. For comparison, the normal distribution has a kurtosis of 3, so this distribution has fewer outliers than the normal distribution.

Feature describing variation in lumen arrangement and arrangement

The single feature in this category, kurtosis of graph edge length in the lumen subgraphs, described the architecture of the lumens across the tumor, rather than lumen shape as the previous features did.

Sub-graph feature: Kurtosis of graph edge length

Kurtosis describes how much of a distribution is composed of outliers. Distributions with a high kurtosis have more outliers, producing a distribution with thicker tails, while distributions with a low kurtosis have tails that quickly drop to zero. This measure was applied to the distribution of edge lengths in the sub-graph of gland lumens, the edges being connections between nearby lumens, as illustrated in Figure 8.

In this study, a lower kurtosis of edge length, that is, having fewer edges which were much longer or shorter than other edges in the image, was associated with increased risk of BCR. As may be observed in Figure 9, the low-kurtosis tumors tended to have clusters of densely packed lumen separated by swaths of stromal tissue. These stromal regions were too large to be spanned by the sub-graph edges, and as a result the sub-graphs of these images contained almost exclusively short edges. In contrast, the high-kutosis images had larger glandular regions containing both small and large glands with a mix of dense and sparse gland packing, producing a mix of short and long edges.

Clusters of glands within expanses of stroma are not seen in normal prostate tissue and the existence of these regions may suggest that the tumor has more substantially altered the normal prostate morphology. Similarly, a tumor which consists of tightly packed glands divided by a thick layer of stroma may have invaded a previously purely stromal region. Either of these may be signs of an aggressive tumor which is more likely to recur.

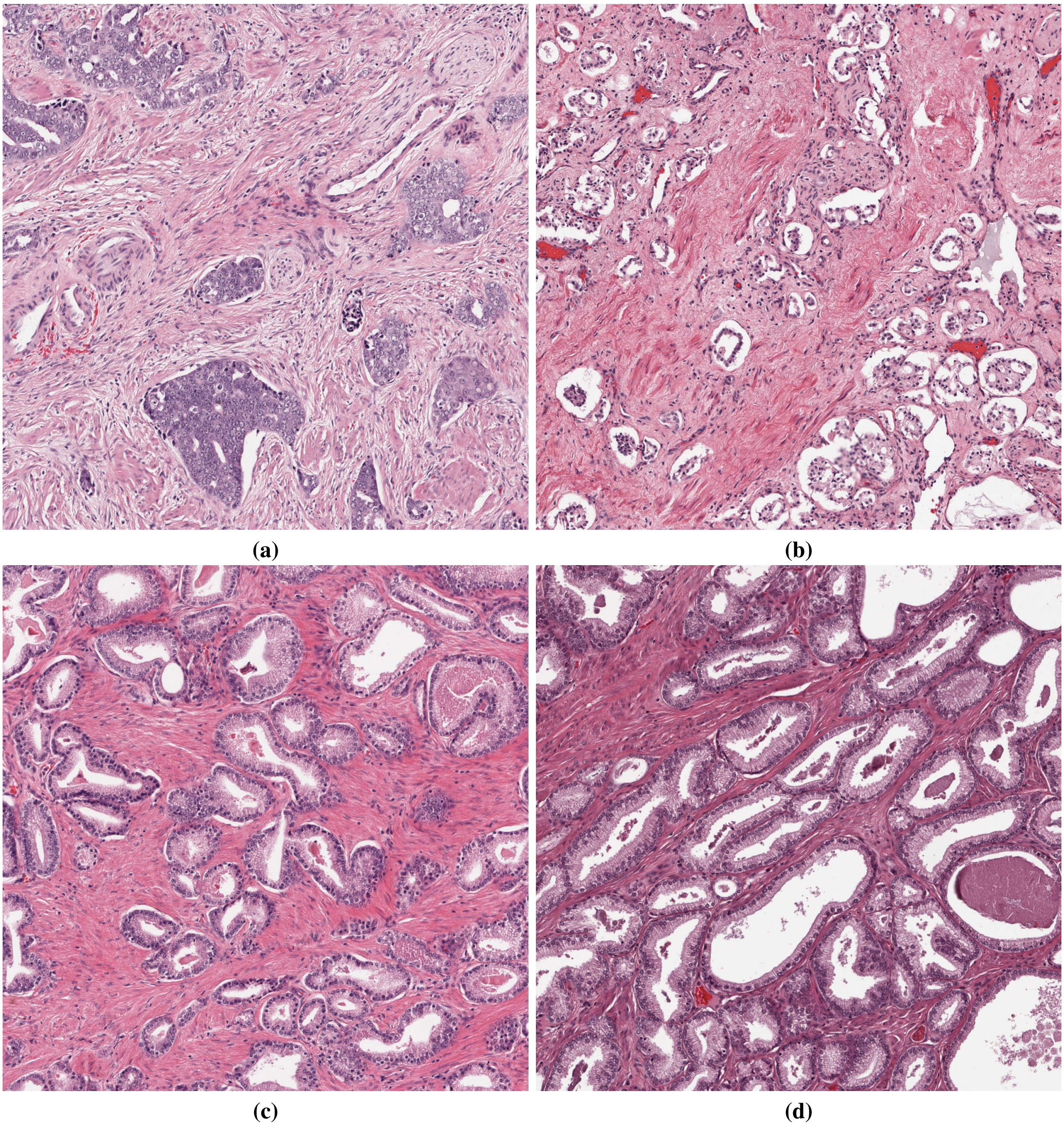

Supplementary Figure 9: Selected regions exhibiting (a)(b) low kurtosis of lumen sub-graph edge length and (c)(d) high kurtosis of lumen sub-graph edge length. Low kurtosis corresponds to uniformly spaced lumen while high kurtosis is associated with irregular lumen spacing. Low kurtosis, as seen in (a) and (b), was associated with increased risk of BCR.

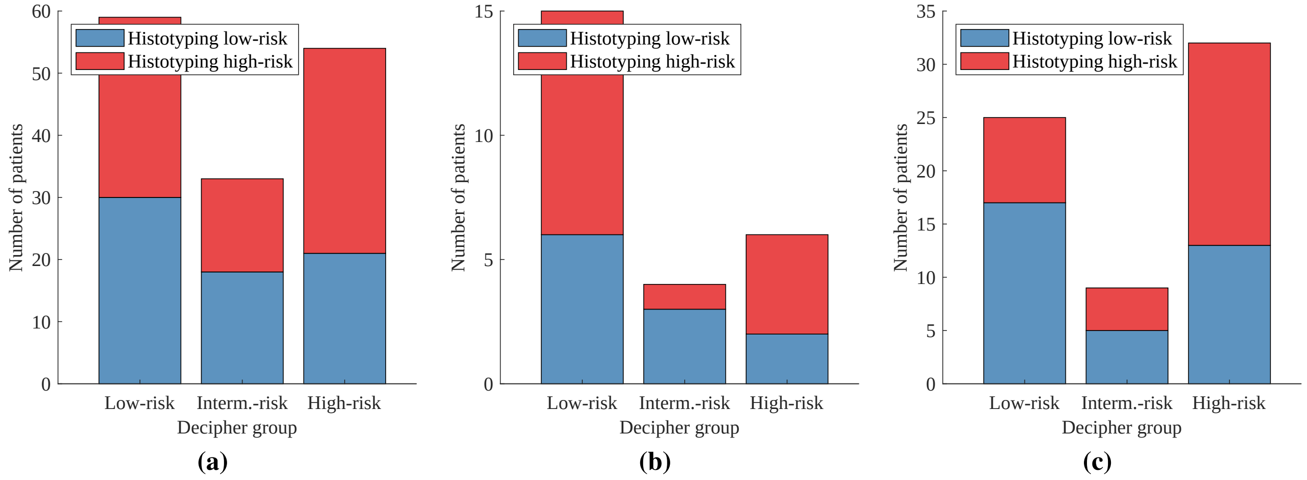

Supplementary Figure 10: Concordance between Decipher and Histotyping risk categories in patients from (a) University of Pennsylvania, (b) Mount Sinai, and (c) the Cleveland Clinic

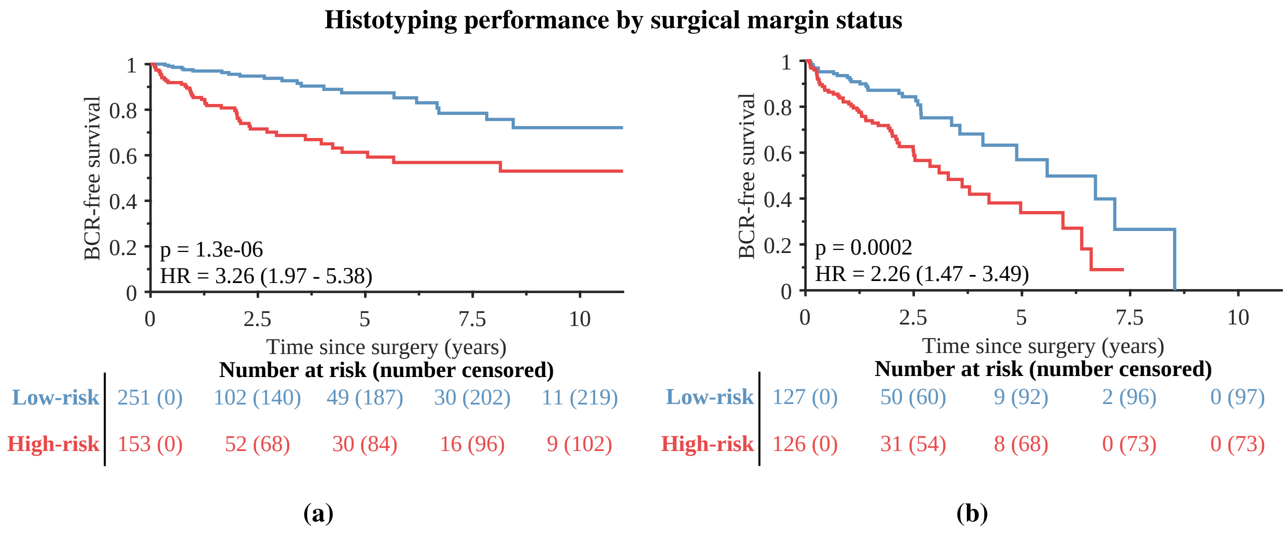

Supplementary Figure 11: Kaplan-Meier BCR-free survival plots of patients categorized as (blue) low-risk and (red) high-risk in (a) surgical margin negative and (b) surgical margin positive patients of the validation set.

Histotyping performance by age

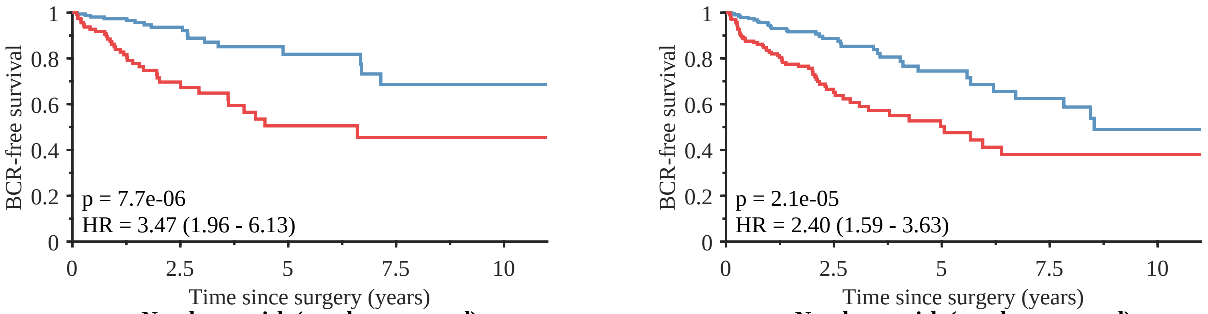

Number at risk (number censored) (a)

**Low-risk**	168 (0)	64 (96)	24 (130)	14 (137)	5 (146)
**High-risk**	113 (0)	30 (57)	16 (64)	7 (72)	4 (75)

(a)

Number at risk (number censored) (b)

**Low-risk**	205 (0)	83 (105)	32 (147)	17 (158)	6 (166)
**High-risk**	165 (0)	50 (69)	20 (91)	7 (100)	5 (102)

(b)

Supplementary Figure 12: Kaplan-Meier BCR-free survival plots of patients categorized as (blue) low-risk and (red) high-risk in (a) patients aged younger than 60 at time of surgery and (b) patients 60 or older at time of surgery of the validation set.

Histotyping performance by pre-operative PSA

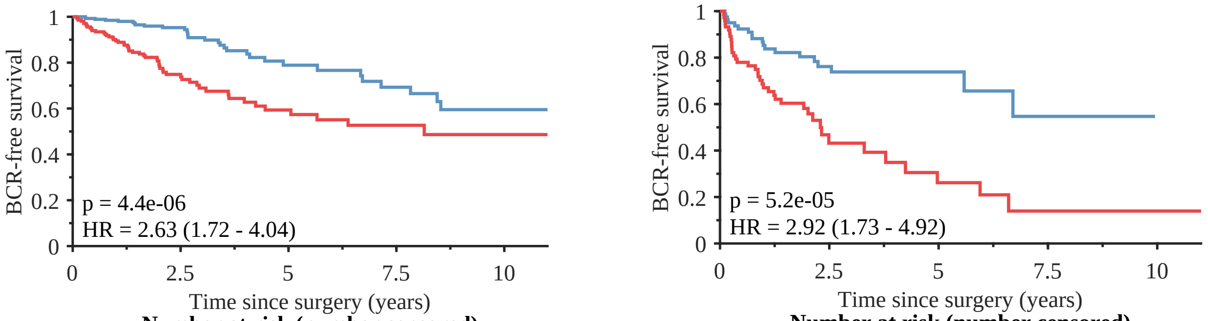

Number at risk (number censored) (a)

**Low-risk**	300 (0)	117 (173)	43 (233)	26 (246)	9 (260)
**High-risk**	208 (0)	69 (99)	31 (126)	14 (140)	7 (146)

(a)

Number at risk (number censored) (b)

**Low-risk**	80 (0)	34 (30)	11 (52)	4 (57)	0 (61)
**High-risk**	74 (0)	12 (29)	6 (31)	2 (33)	2 (33)

(b)

Supplementary Figure 13: Kaplan-Meier BCR-free survival plots of patients categorized as (blue) low-risk and (red) high-risk in (a) patients with a pre-operative PSA level less than 10 and (b) patients with a pre-operative PSA greater than or equal to 10 of the validation set.

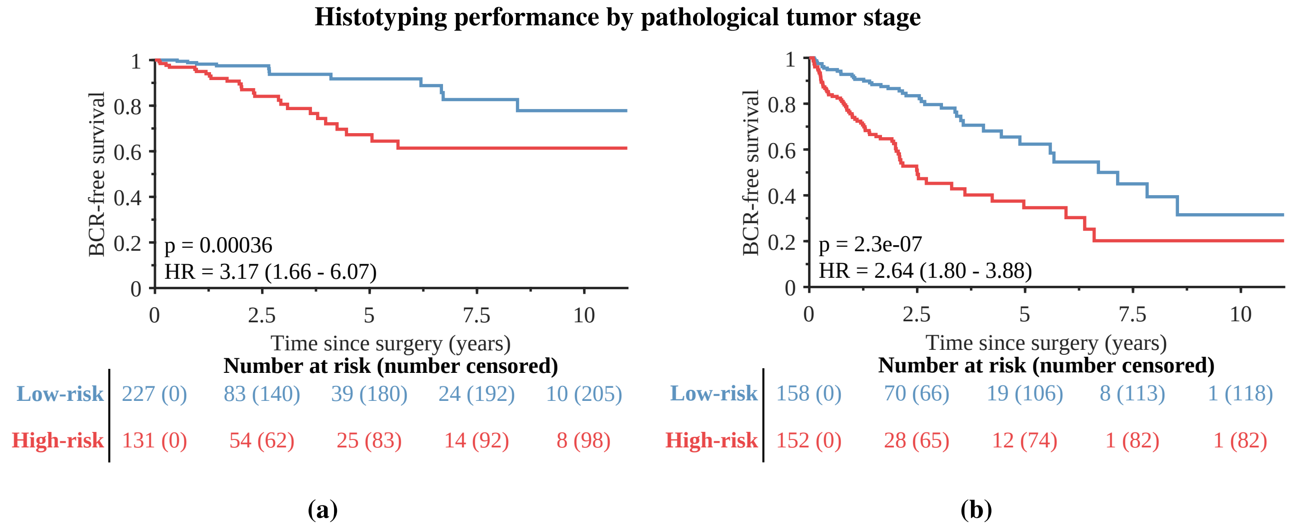

Supplementary Figure 14: Kaplan-Meier BCR-free survival plots of patients categorized as (blue) low-risk and (red) high-risk in (a) pT2 (b) pT3 patients of the validation set.

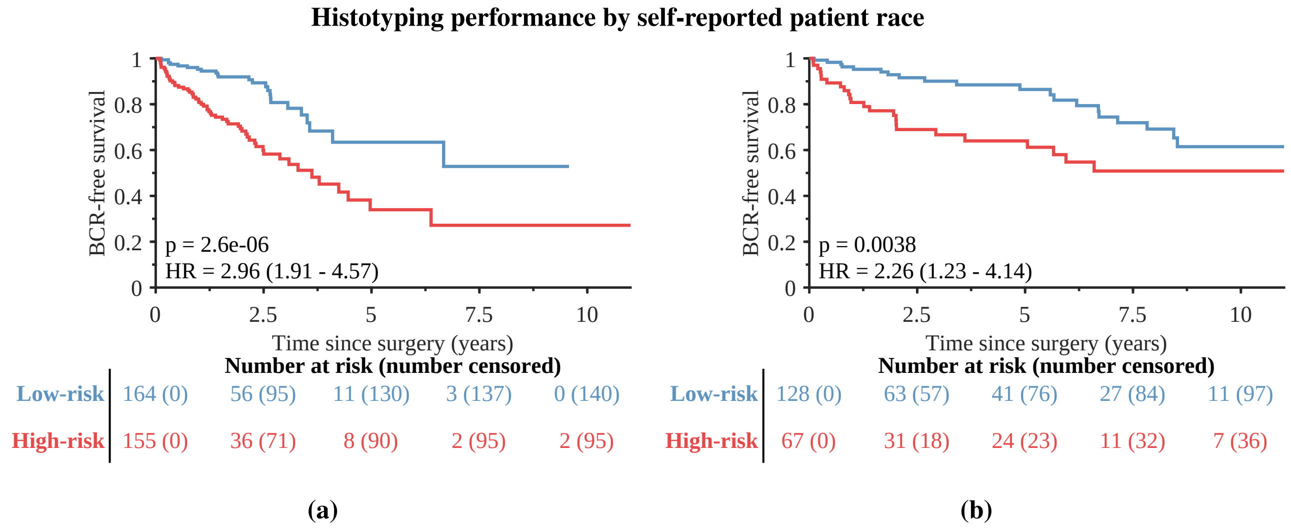

Supplementary Figure 15: Kaplan-Meier BCR-free survival plots of patients categorized as (blue) low-risk and (red) high-risk in (a) Caucasian-American and (b) African-American patients of the validation set.

Histotyping performance by Gleason grade group

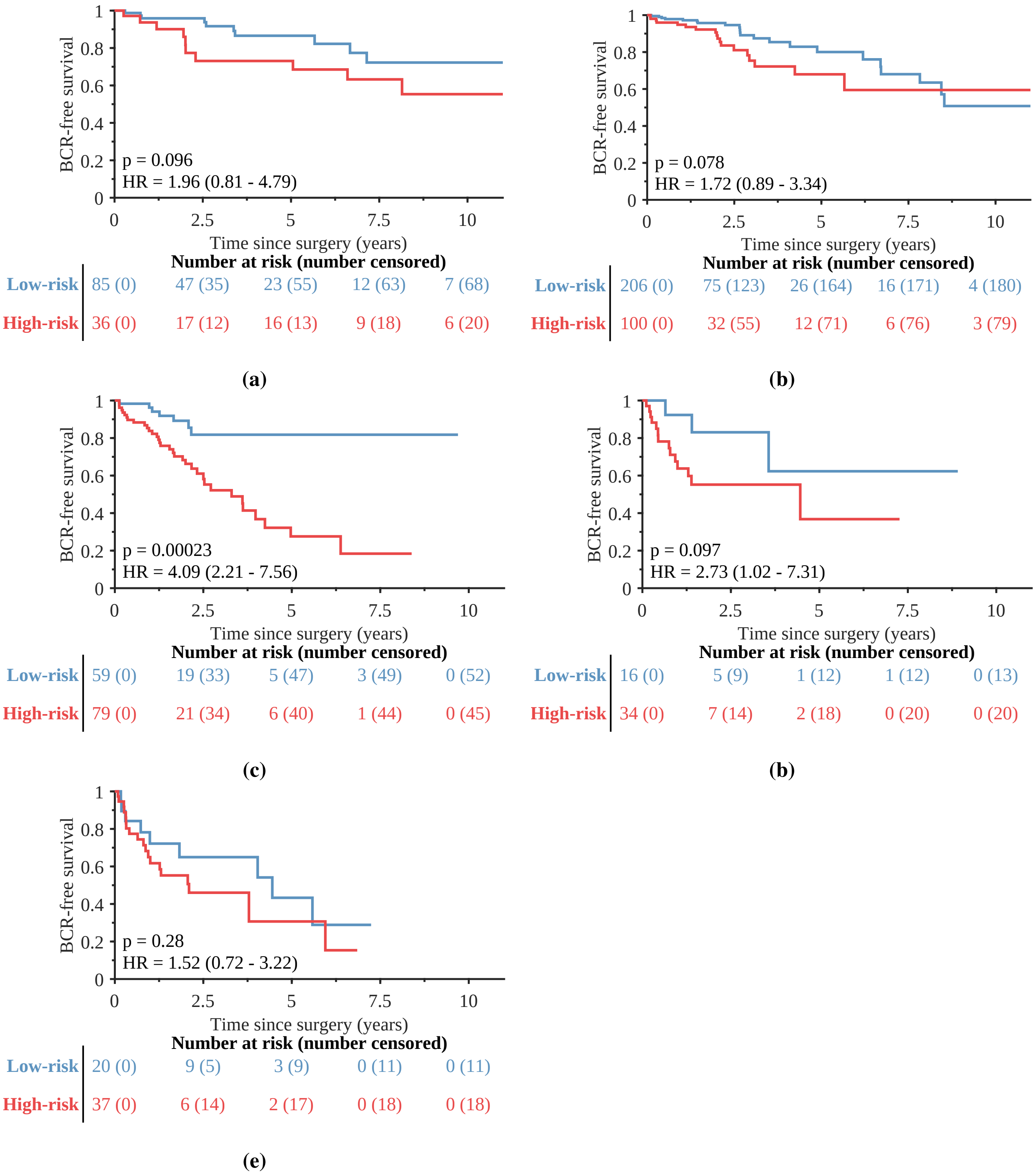

Supplementary Figure 16: Kaplan-Meier BCR-free survival plots of patients categorized as (blue) low-risk and (red) high-risk in Gleason grade group (a) 1, (b) 2, (c) 3, (d) 4, and (e) 5 patients of the validation set.

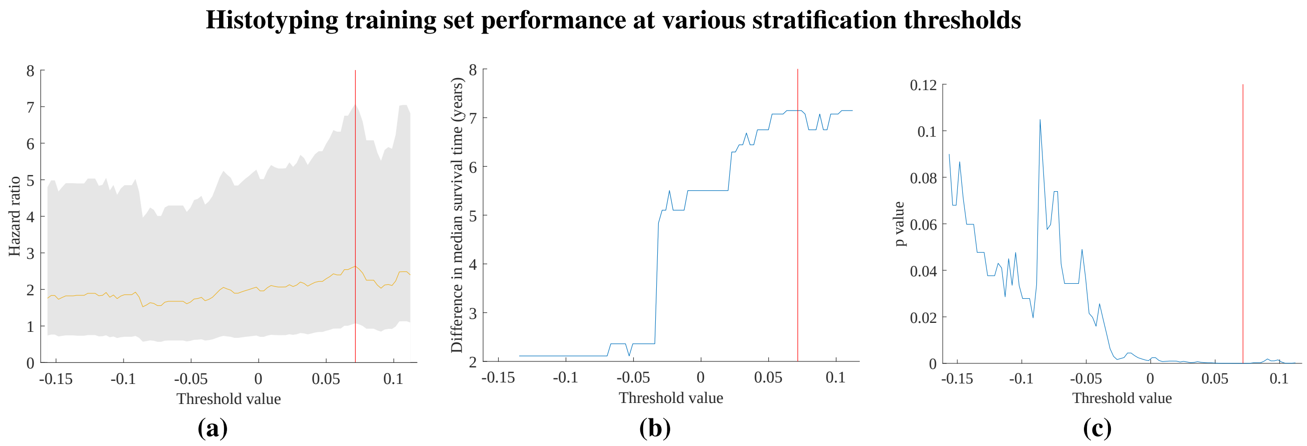

Supplementary Figure 17: Histotyping (a) hazard ratio (yellow line) and 95% confidence interval (shaded area), (b) difference in median survival time, and (c) logrank p-value in the training set at various stratification thresholds. The threshold value used is marked by a vertical red line.

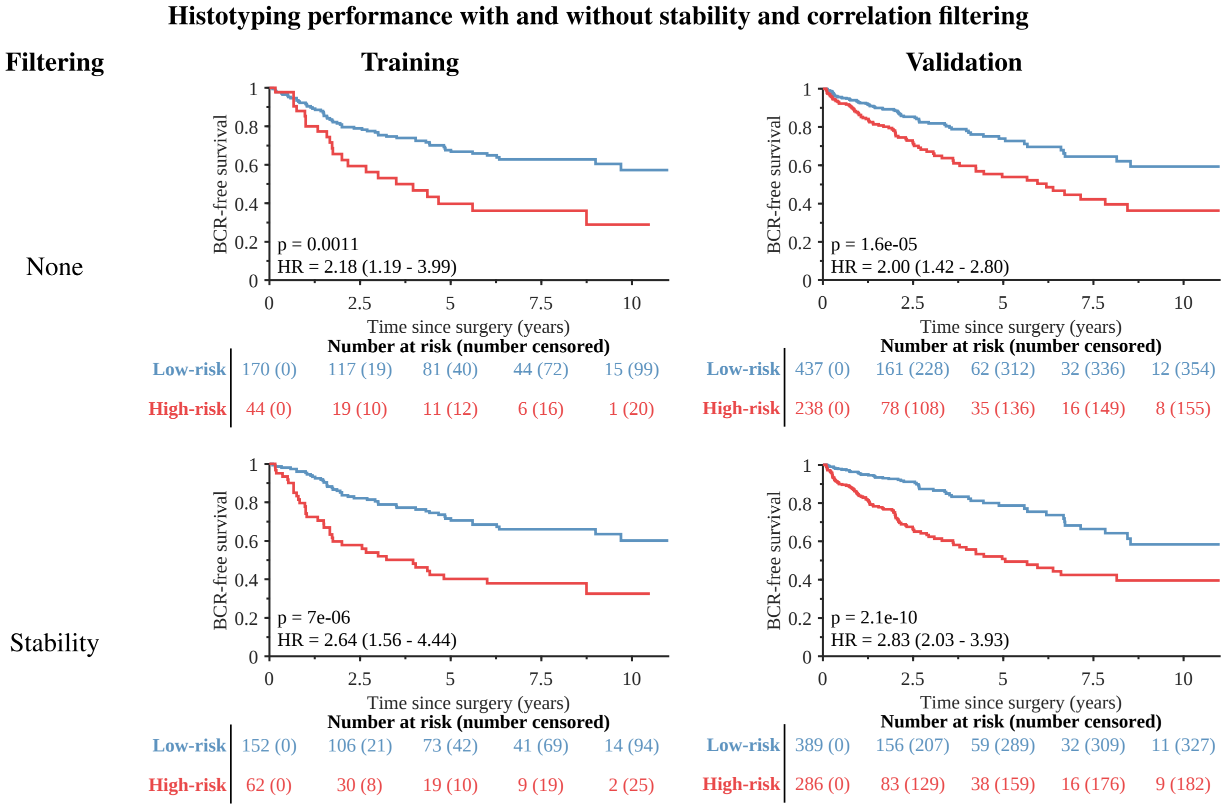

Supplementary Figure 18: Histotyping performance in the (left) training and (right) validation sets with and without feature stability filtering. Filtering parameters were optimized on the training set.

References

[1] P. Bankhead, M. B. Loughrey, J. A. Fernández, Y. Dombrowski, D. G. McArt, P. D. Dunne, S. McQuaid, R. T. Gray, L. J. Murray, H. G. Coleman, J. A. James, M. Salto-Tellez, P. W. Hamilton, QuPath: Open source software for digital pathology image analysis, Scientific Reports 7 (1) (dec 2017). doi:10.1038/s41598-017-17204-5.

[2] O. Ronneberger, P. Fischer, T. Brox, U-net: Convolutional networks for biomedical image segmentation, in: Lecture Notes in Computer Science, Springer International Publishing, 2015, pp. 234–241 (2015). doi:10.1007/978-3-319-24574-4-28.

[3] P. Leo, R. Elliott, N. N. C. Shih, S. Gupta, M. Feldman, A. Madabhushi, Stable and discriminating features are predictive of cancer presence and gleason grade in radical prostatectomy specimens: a multi-site study, Scientific Reports 8 (1) (oct 2018). doi:10.1038/s41598-018-33026-5.

[4] P. Leo, G. Lee, N. N. C. Shih, R. Elliott, M. D. Feldman, A. Madabhushi, Evaluating stability of histomorphometric features across scanner and staining variations: prostate cancer diagnosis from whole slide images, Journal of Medical Imaging 3 (4) (2016) 047502 (2016). doi:10.1117/1.JMI.3.4.047502.
URL `http://dx.doi.org/10.1117/1.JMI.3.4.047502`

[5] D. F. Gleason, G. T. Mellinger, Prediction of prognosis for prostatic adenocarcinoma by combined histological grading and clinical staging., The Journal of Urology 111 (1) (1974) 58–64 (1974).

[6] J. I. Epstein, L. Egevad, M. B. Amin, B. Delahunt, J. R. Srigley, P. A. Humphrey, The 2014 international society of urological pathology (ISUP) consensus conference on gleason grading of prostatic carcinoma, The American Journal of Surgical Pathology (2015) 1 (oct 2015). doi:10.1097/pas.0000000000000530.

[7] M.-K. Hu, Visual pattern recognition by moment invariants, IEEE Transactions on Information Theory 8 (2) (1962) 179–187 (feb 1962). doi:10.1109/tit.1962.1057692.

[8] V. Gudivada, V. Raghavan, Content based image retrieval systems, Computer 28 (9) (1995) 18–22 (1995). doi:10.1109/2.410145.

[9] C. T. Zahn, R. Z. Roskies, Fourier descriptors for plane closed curves, IEEE Transactions on Computers C-21 (3) (1972) 269–281 (mar 1972). doi:10.1109/tc.1972.5008949.

[10] D. Zhang, G. Lu, Shape-based image retrieval using generic fourier descriptor, Signal Processing: Image Communication 17 (10) (2002) 825–848 (nov 2002). doi:10.1016/s0923-5965(02)00084-x.

Supplementary Table 2: Performance metrics for all 242 extracted features

Feature name	p-value (Cox regression)	Hazard ratio (Cox regression)	Instability score
Voronoi: Area std.	0.31	1.08	0.80
Voronoi: Area average	0.22	1.09	0.68
Voronoi: Area 5% / 95%	0.77	1.03	0.10
Voronoi: Area disorder	0.97	1.00	0.46
Voronoi: Perimeter std.	0.18	1.11	0.77
Voronoi: Perimeter average	0.23	1.11	0.67
Voronoi: Perimeter 5% / 95%	0.59	0.94	0.15
Voronoi: Perimeter disorder	0.28	1.12	0.41
Voronoi: Chord std.	0.19	1.11	0.80
Voronoi: Chord average	0.20	1.11	0.67
Voronoi: Chord 5% / 95%	0.19	0.87	0.34
Voronoi: Chord disorder	0.39	1.10	0.44
Delaunay: Side length 5% / 95%	0.62	0.95	0.02
Delaunay: Side length std.	0.29	1.10	0.80
Delaunay: Side length average	0.30	1.09	0.67
Delaunay: Side length disorder	0.39	1.10	0.53
Delaunay: Triangle area 5% / 95%	0.89	1.02	0.04
Delaunay: Triangle area std.	0.21	1.09	0.82
Delaunay: Triangle area average	0.23	1.09	0.71
Delaunay: Triangle area disorder	0.31	1.12	0.51
MST: Edge length average	0.49	1.06	0.67
MST: Edge length std.	0.37	1.08	0.67
MST: Edge length 5% / 95%	0.29	1.13	0.10
MST: Edge length disorder	0.65	1.05	0.28
Arch: Area of polygons	0.99	1.00	0.80
Arch: Number of polygons	0.83	1.03	0.66

Arch: Density of polygons	0.48	0.92	0.87
Arch: Average distance to 3 nearest neighbors	0.59	1.06	0.78
Arch: Average distance to 5 nearest neighbors	0.47	1.07	0.75
Arch: Average distance to 7 nearest neighbors	0.42	1.08	0.74
Arch: Std. distance to 3 nearest neighbors	0.80	1.03	0.79
Arch: Std. distance to 5 nearest neighbors	0.73	1.04	0.72
Arch: Std. distance to 7 nearest neighbors	0.71	1.04	0.69
Arch: Disorder of distance to 3 nearest neighbors	0.49	0.92	0.25
Arch: Disorder of distance to 5 nearest neighbors	0.74	0.96	0.28
Arch: Disorder of distance to 7 nearest neighbors	0.90	0.99	0.33
Arch: Avg. nearest neighbors in a 10 pixel radius	0.31	1.13	0.67
Arch: Avg. nearest neighbors in a 20 pixel radius	0.54	1.08	0.68
Arch: Avg. nearest neighbors in a 30 pixel radius	0.45	1.09	0.67
Arch: Avg. nearest neighbors in a 40 pixel radius	0.43	1.10	0.67
Arch: Avg. nearest neighbors in a 50 pixel radius	0.45	1.09	0.67
Arch: Std. neighbors in 10 pixel radius	0.96	1.01	0.56
Arch: Std. neighbors in 20 pixel radius	0.09	1.22	0.52
Arch: Std. neighbors in 30 pixel radius	0.03	1.27	0.55
Arch: Std. neighbors in 40 pixel radius	0.07	1.24	0.54
Arch: Std. neighbors in 50 pixel radius	0.07	1.24	0.61
Arch: Disorder neighbors in 10 pixel radius	0.61	0.94	0.77
Arch: Disorder neighbors in 20 pixel radius	0.18	1.16	0.75
Arch: Disorder neighbors in 30 pixel radius	0.07	1.21	0.64
Arch: Disorder neighbors in 40 pixel radius	0.14	1.18	0.58
Arch: Disorder neighbors in 50 pixel radius	0.14	1.18	0.61
Shape: Mean area ratio	0.00	1.38	0.13
Shape: Mean average / max distance	0.01	1.36	0.08
Shape: Mean std. of distance	0.00	0.72	0.19
Shape: Mean variance of distance	0.01	0.74	0.22
Shape: Mean distance ratio	0.31	0.89	0.15
Shape: Mean perimeter ratio	0.10	0.81	0.05
Shape: Mean smoothness	0.04	0.77	0.50
Shape: Mean invariant 1	0.05	0.78	0.35
Shape: Mean invariant 2	0.01	0.68	0.02
Shape: Mean invariant 3	0.22	0.81	0.01
Shape: Mean invariant 4	0.23	0.61	0.08
Shape: Mean invariant 5	0.63	0.06	0.26
Shape: Mean invariant 6	0.45	0.36	0.25
Shape: Mean invariant 7	0.55	0.89	0.01
Shape: Mean fractal dimension	0.62	1.06	0.01
Shape: Mean Fourier 1	0.16	1.17	0.00
Shape: Mean Fourier 2	0.72	0.97	0.17
Shape: Mean Fourier 3	0.08	1.24	0.01
Shape: Mean Fourier 4	0.08	1.39	0.12
Shape: Mean Fourier 5	0.72	0.96	0.06
Shape: Mean Fourier 6	0.76	1.03	0.05
Shape: Mean Fourier 7	0.66	0.96	0.20
Shape: Mean Fourier 8	0.51	0.94	0.18
Shape: Mean Fourier 9	0.42	1.11	0.25
Shape: Mean Fourier 10	0.60	1.06	0.22
Shape: Std. area ratio	0.27	1.14	0.17
Shape: Std. average / max distance	0.53	0.93	0.03

Shape: Std. Std. of distance	0.29	1.14	0.11
Shape: Std. variance of distance	0.69	0.96	0.50
Shape: Std. distance ratio	0.04	0.78	0.04
Shape: Std. perimeter ratio	0.31	0.88	0.00
Shape: Std. smoothness	0.02	0.68	0.20
Shape: Std. invariant 1	0.01	0.66	0.03
Shape: Std. invariant 2	0.06	0.65	0.06
Shape: Std. invariant 3	0.38	0.87	0.16
Shape: Std. invariant 4	0.20	0.49	0.37
Shape: Std. invariant 5	0.51	0.10	0.39
Shape: Std. invariant 6	0.38	0.37	0.37
Shape: Std. invariant 7	0.26	0.17	0.37
Shape: Std. fractal dimension	0.01	0.73	0.22
Shape: Std. Fourier 1	0.03	0.73	0.82
Shape: Std. Fourier 2	0.12	0.80	0.71
Shape: Std. Fourier 3	0.13	0.82	0.72
Shape: Std. Fourier 4	0.13	0.82	0.71
Shape: Std. Fourier 5	0.07	0.78	0.70
Shape: Std. Fourier 6	0.04	0.74	0.60
Shape: Std. Fourier 7	0.45	0.91	0.74
Shape: Std. Fourier 8	0.05	0.77	0.76
Shape: Std. Fourier 9	0.04	0.74	0.68
Shape: Std. Fourier 10	0.23	0.86	0.72
Shape: Median area ratio	0.00	1.40	0.07
Shape: Median average / max distance	0.00	1.42	0.05
Shape: Median Std. of distance	0.00	0.72	0.18
Shape: Median variance of distance	0.01	0.74	0.18
Shape: Median distance ratio	0.12	0.84	0.33
Shape: Median perimeter ratio	0.05	0.78	0.20
Shape: Median smoothness	0.47	0.92	0.11
Shape: Median invariant 1	0.20	0.86	0.08
Shape: Median invariant 2	0.04	0.77	0.14
Shape: Median invariant 3	0.19	0.86	0.29
Shape: Median invariant 4	0.34	0.89	0.26
Shape: Median invariant 5	0.63	0.93	0.04
Shape: Median invariant 6	0.57	0.86	0.14
Shape: Median invariant 7	0.19	0.91	0.00
Shape: Median fractal dimension	0.17	1.17	0.00
Shape: Median Fourier 1	0.53	1.07	0.02
Shape: Median Fourier 2	0.26	1.15	0.72
Shape: Median Fourier 3	0.12	1.36	0.35
Shape: Median Fourier 4	0.11	1.30	0.63
Shape: Median Fourier 5	0.37	1.13	0.76
Shape: Median Fourier 6	0.45	1.09	0.68
Shape: Median Fourier 7	0.76	0.97	0.65
Shape: Median Fourier 8	0.18	1.17	0.66
Shape: Median Fourier 9	0.64	1.06	0.69
Shape: Median Fourier 10	0.38	1.13	0.83
Shape: 5% / 95% area ratio	0.20	1.15	0.24
Shape: 5% / 95% average / max distance	0.39	1.10	0.00
Shape: 5% / 95% std. of distance	0.01	0.77	0.15
Shape: 5% / 95% variance of distance	0.03	0.76	0.16

Shape: 5% / 95% distance ratio	0.08	1.22	0.03
Shape: 5% / 95% perimeter ratio	0.02	1.29	0.02
Shape: 5% / 95% smoothness	0.00	1.49	0.10
Shape: 5% / 95% invariant 1	0.03	1.28	0.00
Shape: 5% / 95% invariant 2	0.11	1.12	0.02
Shape: 5% / 95% invariant 3	1.00	1.00	0.00
Shape: 5% / 95% invariant 4	1.00	1.00	0.00
Shape: 5% / 95% invariant 5	0.05	0.85	0.10
Shape: 5% / 95% invariant 6	0.92	1.01	0.14
Shape: 5% / 95% invariant 7	0.13	0.90	0.01
Shape: 5% / 95% fractal dimension	0.00	1.39	0.12
Shape: 5% / 95% Fourier 1	0.15	2.29	0.01
Shape: 5% / 95% Fourier 2	0.63	0.96	0.02
Shape: 5% / 95% Fourier 3	0.86	1.02	0.05
Shape: 5% / 95% Fourier 4	0.88	1.02	0.04
Shape: 5% / 95% Fourier 5	0.58	0.95	0.03
Shape: 5% / 95% Fourier 6	0.01	0.76	0.00
Shape: 5% / 95% Fourier 7	0.16	0.88	0.14
Shape: 5% / 95% Fourier 8	0.22	0.90	0.11
Shape: 5% / 95% Fourier 9	0.00	0.72	0.06
Shape: 5% / 95% Fourier 10	0.01	0.81	0.11
CGT: Mean tensor contrast energy	0.76	1.03	0.35
CGT: Std. tensor contrast energy	0.40	1.09	0.67
CGT: Range tensor contrast energy	0.76	1.03	0.61
CGT: Mean tensor contrast inverse	0.93	1.01	0.68
CGT: Std. tensor contrast inverse	0.50	1.07	0.64
CGT: Range tensor contrast inverse	0.93	1.01	0.69
CGT: Mean tensor contrast average	0.75	1.03	0.53
CGT: Std. tensor contrast average	0.44	1.08	0.65
CGT: Range tensor contrast average	0.80	1.03	0.64
CGT: Mean tensor contrast var	0.79	1.03	0.05
CGT: Std. tensor contrast var	0.44	1.08	0.67
CGT: Range tensor contrast var	0.44	1.09	0.70
CGT: Mean tensor contrast entropy	0.90	0.99	0.67
CGT: Std. tensor contrast entropy	0.57	1.06	0.67
CGT: Range tensor contrast entropy	0.98	1.00	0.58
CGT: Mean tensor intensity average	0.84	1.02	0.08
CGT: Std. tensor intensity average	0.54	1.06	0.60
CGT: Range tensor intensity average	0.58	1.06	0.69
CGT: Mean tensor intensity variance	0.73	1.04	0.14
CGT: Std. tensor intensity variance	0.36	1.09	0.67
CGT: Range tensor intensity variance	0.47	1.08	0.76
CGT: Mean tensor intensity entropy	0.90	0.99	0.67
CGT: Std. tensor intensity entropy	0.58	1.06	0.67
CGT: Range tensor intensity entropy	0.98	1.00	0.65
CGT: Mean tensor entropy	0.94	0.99	0.68
CGT: Std. tensor entropy	0.63	1.05	0.59
CGT: Range tensor entropy	0.87	0.98	0.63
CGT: Mean tensor energy	0.76	1.03	0.67
CGT: Std. tensor energy	0.50	1.07	0.67
CGT: Range tensor energy	0.82	1.03	0.61
CGT: Mean tensor correlation	0.93	0.99	0.26

CGT: Std. tensor correlation	0.56	1.06	0.61
CGT: Range tensor correlation	0.71	1.04	0.62
CGT: Mean tensor information measure 1	0.96	1.01	0.56
CGT: Std. tensor information measure 1	0.87	1.02	0.47
CGT: Range tensor information measure 1	0.99	1.00	0.67
CGT: Mean tensor information measure 2	0.95	1.01	0.55
CGT: Std. tensor information measure 2	0.68	1.04	0.65
CGT: Range tensor information measure 2	1.00	1.00	0.70
Sub-Graph: Number of nodes	0.83	1.03	0.66
Sub-Graph: Number of edges	0.47	1.09	0.59
Sub-Graph: Average degree	0.42	1.10	0.68
Sub-Graph: Average eccentricity	0.74	1.04	0.70
Sub-Graph: Diameter	0.72	1.04	0.68
Sub-Graph: Radius	0.18	1.14	0.47
Sub-Graph: Average eccentricity 90%	0.80	1.03	0.70
Sub-Graph: Diameter 90%	0.72	1.04	0.70
Sub-Graph: Radius 90%	0.21	1.13	0.46
Sub-Graph: Average path length	0.60	1.06	0.65
Sub-Graph: Clustering coefficient c	0.74	0.97	0.66
Sub-Graph: Clustering coefficient d	0.96	0.99	0.68
Sub-Graph: Clustering coefficient e	0.89	0.99	0.68
Sub-Graph: Number of connected components	0.83	1.03	0.65
Sub-Graph: Giant connected component ratio	0.28	0.90	0.73
Sub-Graph: Average connected component size	0.89	1.02	0.75
Sub-Graph: Number isolated nodes	0.73	1.04	0.85
Sub-Graph: Percentage isolated nodes	0.13	1.10	0.69
Sub-Graph: Number end nodes	0.35	1.09	0.83
Sub-Graph: Percentage end nodes	0.21	1.09	0.69
Sub-Graph: Number central nodes	0.81	1.03	0.50
Sub-Graph: Percentage central nodes	0.43	0.91	0.63
Sub-Graph: Mean edge length	0.61	0.95	0.04
Sub-Graph: Std. edge length	0.05	1.22	0.05
Sub-Graph: Skewness edge length	0.31	1.10	0.20
Sub-Graph: Kurtosis edge length	0.02	0.79	0.07
Haralick: Mean contrast energy	0.19	0.86	0.74
Haralick: Std. contrast energy	0.12	0.83	0.71
Haralick: Mean contrast inverse	0.59	0.94	0.11
Haralick: Std. contrast inverse	0.14	0.81	0.04
Haralick: Mean contrast average	0.19	0.86	0.68
Haralick: Std. contrast average	0.08	0.81	0.67
Haralick: Mean contrast var	0.19	0.85	0.73
Haralick: Std. contrast var	0.13	0.83	0.68
Haralick: Mean contrast entropy	0.99	1.00	0.30
Haralick: Std. contrast entropy	0.26	0.86	0.37
Haralick: Mean intensity average	0.01	1.33	0.87
Haralick: Std. intensity average	0.01	0.73	0.45
Haralick: Mean intensity variance	0.15	0.84	0.72
Haralick: Std. intensity variance	0.16	0.84	0.67
Haralick: Mean intensity entropy	0.77	1.04	0.23
Haralick: Std. intensity entropy	0.31	0.87	0.38
Haralick: Mean entropy	0.52	1.09	0.23
Haralick: Std. entropy	0.32	0.88	0.38

Haralick: Mean energy	0.32	0.84	0.07
Haralick: Std. energy	0.19	0.83	0.31
Haralick: Mean correlation	0.03	0.78	0.08
Haralick: Std. correlation	0.01	0.75	0.41
Haralick: Mean information measure 1	0.39	0.87	0.01
Haralick: Std. information measure 1	0.23	0.85	0.34
Haralick: Mean information measure 2	0.64	1.06	0.19
Haralick: Std. information measure 2	0.32	0.88	0.35
