# Supplementary material for: Computer extracted gland features from H&E predicts prostate cancer recurrence comparably to a genomic companion diagnostic test: a large multi-site study
Source: NPJ Precis Oncol. 2021 May 3;5:35. doi: 10.1038/s41698-021-00174-3 (PMC8093226; doi:10.1038/s41698-021-00174-3)
Supplement: Supplementary file 2 — Reporting Summary [file 41698_2021_174_MOESM2_ESM.pdf]

nature research

Corresponding author(s): Anant Madabhushi

Last updated by author(s): Dec 10, 2020

Reporting Summary

Nature Research wishes to improve the reproducibility of the work that we publish. This form provides structure for consistency and transparency in reporting. For further information on Nature Research policies, see our Editorial Policies and the Editorial Policy Checklist.

Statistics

For all statistical analyses, confirm that the following items are present in the figure legend, table legend, main text, or Methods section.

n/a	Confirmed	
☐	☒	The exact sample size (*n*) for each experimental group/condition, given as a discrete number and unit of measurement
☐	☒	A statement on whether measurements were taken from distinct samples or whether the same sample was measured repeatedly
☐	☒	The statistical test(s) used AND whether they are one- or two-sided<br>*Only common tests should be described solely by name; describe more complex techniques in the Methods section.*
☐	☒	A description of all covariates tested
☐	☒	A description of any assumptions or corrections, such as tests of normality and adjustment for multiple comparisons
☐	☒	A full description of the statistical parameters including central tendency (e.g. means) or other basic estimates (e.g. regression coefficient) AND variation (e.g. standard deviation) or associated estimates of uncertainty (e.g. confidence intervals)
☐	☒	For null hypothesis testing, the test statistic (e.g. *F*, *t*, *r*) with confidence intervals, effect sizes, degrees of freedom and *P* value noted<br>*Give P values as exact values whenever suitable.*
☒	☐	For Bayesian analysis, information on the choice of priors and Markov chain Monte Carlo settings
☒	☐	For hierarchical and complex designs, identification of the appropriate level for tests and full reporting of outcomes
☐	☒	Estimates of effect sizes (e.g. Cohen's *d*, Pearson's *r*), indicating how they were calculated

Our web collection on statistics for biologists contains articles on many of the points above.

Software and code

Policy information about availability of computer code

Data collection	Aperio Imagescope was used to perform annotations of tumor regions in this study. QuPath was used to annotate gland lumen boundaries.
Data analysis	In-house code used for this project consists of feature extraction code available at https://github.com/pjl54/matlab-feature-extraction.

For manuscripts utilizing custom algorithms or software that are central to the research but not yet described in published literature, software must be made available to editors and reviewers. We strongly encourage code deposition in a community repository (e.g. GitHub). See the Nature Research guidelines for submitting code & software for further information.

Data

Policy information about availability of data

All manuscripts must include a data availability statement. This statement should provide the following information, where applicable:
- Accession codes, unique identifiers, or web links for publicly available datasets
- A list of figures that have associated raw data
- A description of any restrictions on data availability

Images and patient data used in this study, other than the publicly available TCGA datasets, are governed by material transfer agreements that preclude the sharing of this data without permission from the original institution. Requests for this data may be addressed to the corresponding author.

Field-specific reporting

Please select the one below that is the best fit for your research. If you are not sure, read the appropriate sections before making your selection.

☒ Life sciences ☐ Behavioural & social sciences ☐ Ecological, evolutionary & environmental sciences

For a reference copy of the document with all sections, see nature.com/documents/nr-reporting-summary-flat.pdf

Life sciences study design

All studies must disclose on these points even when the disclosure is negative.

Sample size	Sample size was not predetermined as cohorts of patients were selected at participating institutions based on chart review. The number of patients (675) and events (148) in the validation set far exceeded the number of covariates in the model (6), lending confidence in the size of the study set.
Data exclusions	Patients were excluded if biochemical recurrence outcome information was not available, as that was the endpoint in this study, or if the patient diagnostic slide could not be located or scanned, as that would prevent analysis of that slide.
Replication	Experiments in this study checked these findings for reproducibility across variation in sample selection and batch effects and found no evidence that replication of this study would be affected by these factors.
Randomization	Participants were not randomized, as no intervention was performed in this study. Patients were allocated to training or testing sets based on the site from which the patient originated.
Blinding	Investigators were not blinded as this retrospective study did not include an intervention to be blinded to.

Reporting for specific materials, systems and methods

We require information from authors about some types of materials, experimental systems and methods used in many studies. Here, indicate whether each material, system or method listed is relevant to your study. If you are not sure if a list item applies to your research, read the appropriate section before selecting a response.

Materials & experimental systems

n/a	Involved in the study	
☒	☐	Antibodies
☒	☐	Eukaryotic cell lines
☒	☐	Palaeontology and archaeology
☒	☐	Animals and other organisms
☒	☐	Human research participants
☒	☐	Clinical data
☒	☐	Dual use research of concern

Methods

n/a	Involved in the study	
☒	☐	ChIP-seq
☒	☐	Flow cytometry
☒	☐	MRI-based neuroimaging
